# Versatile Bispidine‐Based Bifunctional Chelators for ^64^Cu^II^‐Labelling of Biomolecules

**DOI:** 10.1002/chem.201904654

**Published:** 2020-01-09

**Authors:** Garima Singh, Kristof Zarschler, Sebastian Hunoldt, Irma Ivette Santana Martínez, Carmen L. Ruehl, Madlen Matterna, Ralf Bergmann, Domokos Máthé, Nikolett Hegedüs, Michael Bachmann, Peter Comba, Holger Stephan

**Affiliations:** ^1^ Helmholtz-Zentrum Dresden-Rossendorf Institute of Radiopharmaceutical Cancer Research Bautzner Landstrasse 400 01328 Dresden Germany; ^2^ Anorganisch-Chemisches Institut INF 270 Universität Heidelberg 69120 Heidelberg Germany; ^3^ Department of Biophysics and Radiation Biology Semmelweis University 1094 Budapest Hungary; ^4^ CROmed Translational Research Centers Ltd. 1047 Budapest Hungary

**Keywords:** bifunctional chelators, bispidines, EGFR, imaging, site-specific labelling

## Abstract

Bifunctional chelators as parts of modular metal‐based radiopharmaceuticals are responsible for stable complexation of the radiometal ion and for covalent linkage between the complex and the targeting vector. To avoid loss of complex stability, the bioconjugation strategy should not interfere with the radiometal chelation by occupying coordinating groups. The C9 position of the very stable Cu^II^ chelator 3,7‐diazabicyclo[3.3.1]nonane (bispidine) is virtually predestined to introduce functional groups for facile bioconjugation as this functionalisation does not disturb the metal binding centre. We describe the preparation and characterisation of a set of novel bispidine derivatives equipped with suitable functional groups for diverse bioconjugation reactions, including common amine coupling strategies (bispidine‐isothiocyanate) and the Cu‐free strain‐promoted alkyne–azide cycloaddition. We demonstrate their functionality and versatility in an exemplary way by conjugation to an antibody‐based biomolecule and validate the obtained conjugate in vitro and in vivo.

## Graphical abstract

**Ready for bioconjugation**: Modification of the bispidine backbone at the C9‐position via an ether linkage provides a set of suitable bifunctional chelators ready for incorporating into targeting moieties and ^64^Cu^II^‐labelling. The usefulness of these bifunctional chelators for targeted positron emission tomography (PET) imaging is demonstrated in vivo.

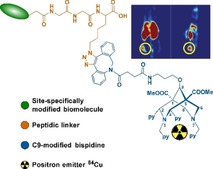

## Introduction

The design of tailor‐made bifunctional chelating agents (BFCs) for radioactive metal ions for nuclear medical applications as well as acquisition of reliable information about the biodistribution of biological objects/biomaterials represents an intensive and rapidly developing field of research.^1^ For theranostic applications (simultaneous therapeutic and diagnostic competencies), BFCs that form very stable complexes with radiometal ions and also allow the simple introduction of vector molecules for pharmaceutical targeting under physiological conditions are of special interest. Bispidine derivatives are such multifunctional ligands with interesting pharmacological1d and metal binding properties.1c, 2 The preorganised and for Cu^II^ complementary geometry of bispidines, adopting a double‐chair conformation and *endo–endo* configuration of the C2/C4 substituents, leads to metal complexes with high thermodynamic stability and kinetic inertness. In recent years, ^64^Cu^II^‐labelled bispidines have gained importance as imaging agents for positron emission tomography (PET).^3^ For this purpose a variety of bispidine ligands bearing in particular pendant pyridine3a, 3f but also imidazole,^4^ pyridazine,3e picolinic acid,^5^ oxine,^6^ and phosphonate^7^ groups as well as bispidine dioxotetraaza macrocycycles3d are available.

The bispidine scaffold also offers the possibility of incorporating fluorescent molecules^8^ for optical imaging as well as providing a site for the attachment of biological vector molecules, such as peptides and biotin.3f, 3g, 7a, 8b With respect to a minor steric influence on the metal binding centre, the C9‐position of bispidines is particularly well suited for the introduction of biomolecules. However, this position is relatively chemically inert. Recently, we have reported the synthesis of a bispidine carbonate that easily allows the formation of carbamates using amine‐functionalised molecules.8b A relevant bispidine‐BODIPY (boron‐dipyrromethene) urethane derivative was sufficiently stable in vitro. An alternative synthesis strategy for the preparation of chemically more stable ether‐linked bispidine derivatives is the reductive alkylation of bispidoles. So far, there is only one example in the literature, namely the preparation of 9‐methoxy and 9‐fluorodecyloxy bispidine derivatives.^9^

**Scheme 1:**
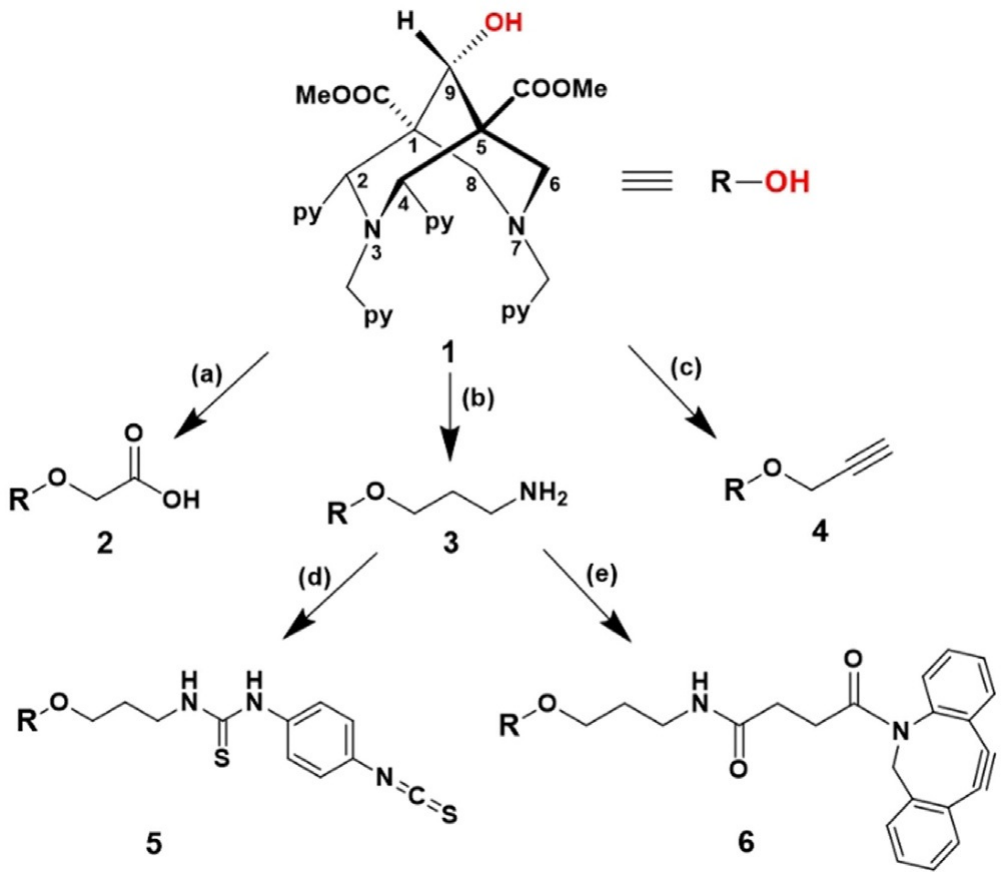
Synthetic approaches to bispidine‐acetic acid **2**, bispidine‐amine **3**, bispidine‐alkyne **4**, bispidine‐isothiocyanate **5** and bispidine‐DBCO **6** by using the bispidine‐9‐ol **1** as the starting compound: (a) THF, sodium hydride (NaH), iodoacetic acid, H_2_O, 50 °C, 2 h, yield=8.6 %; (b) (i) Dry THF, sodium hydride (NaH), *tert*‐butyl(3‐bromopropyl)carbamate, sodium hydrogen carbonate (NaHCO_3_), 50 °C, 20 h, yield=35 %, (ii) TFA/DCM, 1:1 (*v*/*v*), RT, 24 h, yield=100 %; (c) Dry THF, sodium hydride (NaH), propargyl bromide, sodium hydrogen carbonate (NaHCO_3_), 50 °C, 3 h, yield=16 %; (d) DCM, TEA, p‐phenylene diisothiocyanate, RT, 16 h, yield=60 %; (e) DCM, DBCO‐NHS ester, TEA, RT, 2 h, yield=56 %.

In this article, we report the synthesis of novel BFCs based on the hexadendate bis(amine)tetrakis(pyridine) bispidine‐9‐ol (**1**) equipped with suitable functionalities for diverse bioconjugation reactions (Scheme 1). Biomolecules possessing amine or carboxylate groups can be coupled to acetic acid‐functionalised **2** and amine‐terminated **3** bispidines, respectively, to produce bioconjugates with standard peptide coupling. The alkyne‐containing bispidine **4** can be used for conjugation to azide‐functionalised biomolecules forming stable triazole rings, exploiting biorthogonal click chemistry. Using **3** as a key intermediate, novel isothiocyanate‐terminated **5** and dibenzocyclooctyne (DBCO)‐functionalised bispidine **6** can be generated. The amine‐reactive derivative **5** can be readily applied for classical protein modification exploiting the reactivity of lysine functionalities present on the protein surface. However, as this bioconjugation strategy is nonspecific, it typically results in a mixture of conjugates labelled to various extents and at different positions. Conjugation to crucial residues next to the antigen‐binding site of antibodies or active sites of enzymes may heavily affect the affinity and immunoreactivity of the former or diminish the activity of the latter. Thus, the conjugates may differ in their enzymatic activities, solubility, charge, pharmacokinetic profile and antigen‐binding characteristics.

In contrast, site‐specific protein modification results in a more homogenous product population with defined and tunable properties. For that reason, we designed the DBCO‐functionalised bispidine **6** applicable in a generic two‐step chemoenzymatic protein modification approach for the generation of highly defined radioimmunotracers as demonstrated herein in an exemplary way for an epidermal growth factor receptor (EGFR)‐specific camelid single‐domain antibody (sdAb) fragment. This protein is in the first place equipped with a unique biorthogonal azide handle in a site‐specific Sortase A‐mediated conjugation. The single‐conjugated immunoconjugate is completed thereafter by a strain‐promoted azide‐alkyne cycloaddition (SPAAC) reaction using the azide‐functionalised intermediate in combination with the DBCO‐containing chelator **6**. The functionality of the obtained conjugate was validated in vitro and its tumour‐targeting capability was evaluated by small animal PET imaging.

## Results and Discussion

### Synthesis of bispidine derivatives

The bispidine‐9‐ol precursor **1** was synthesised through classical double‐Mannich reactions with reported protocols.3f, 10 The reduction of bispidine was carried out with NaBH_4_ in dioxane–water leading exclusively to the *anti*‐product **1** (OH group in the *anti*‐ position to C2/C4 substituents, see the Supporting Information, Figure S1). A range of exploitable organic functionalities were introduced to intermediate **1** to develop novel C9 modified bispidines **2**–**6**. Under basic conditions using sodium hydride, **1** was reacted to iodoacetic acid, *tert*‐butyl(3‐bromopropyl)carbamate and propargyl bromide, respectively, to yield bispidine–acetic acid **2**, bispidine–amine **3** and bispidine–alkyne **4** derivatives. The reactions proceed by the generation of bispidine–alkoxides, undergoing nucleophilic substitution pathways and subsequent elimination of the halide to yield the desired bispidines. The amine‐functionalised bispidine **3** was coupled to *p*‐phenylene diisothiocyanate to produce the isothiocyanate‐functionalised bispidine **5**, forming a thiourea bond. DBCO‐terminated bispidine **6**, on the other hand, was synthesised by a DBCO–NHS ester coupled to **3** by a stable amide linkage. The synthesised ligands were purified by semipreparative reversed phase HPLC. Due to similar polarity and solubility properties of products and byproducts, this method has proven to be the most suitable for the mg‐scale. Bispidines were analysed by ESI‐MS and further characterised by ^1^H, ^13^C NMR and IR spectroscopy (see the Supporting Information, Figure S2–S6). The molecular ion peaks at *m*/*z*: 653.1 (**2**), 652.1 (**3**), 633.4 (**4**), 843.3 (**5**) and 939.1(**6**) confirmed the identities of synthesised ligands. In the ^1^H NMR spectra, the characteristic proton singlet observed between *δ*=4.6–5 ppm confirms the modifications of the C9 carbon atom of **2**, **3**, and **4**. The protons from the propyl chain of **3** appear in the range of *δ*=3.7–1.9 ppm in the ^1^H NMR whereas the ^13^C NMR displays a characteristic signal for C9 at *δ*=72.6 ppm. The ^1^H NMR of **4** shows the methylene protons at *δ*=3.7 ppm positioned next to the alkyne group. The ^13^C NMR of **5** shows aromatic carbon atoms in the range of *δ*=150‐125 ppm and the characteristic resonance for the isothiocyanate carbon was observed at *δ*=182 ppm. The FTIR spectrum of **2** shows the carboxylic acid O−H stretch as a broad band between 3300–2700 cm^−1^ and the C=O stretch between 1720–1690 cm^−1^. The FTIR of **3** displays strong N−H stretching from 3500–2900 cm^−1^ and a weak bending peak at 1126 cm^−1^. The characteristic carbonyl stretching vibrations for esters (O=C−O) at C1 and C5 is positioned at 1727 cm^−1^ whereas the C−O stretch is at 1200 cm^−1^. The characteristic terminal alkyne with a strong C−H stretch at ≈3000 cm^−1^ and a weak ‐C≡C‐ stretch at ≈2200 cm^−1^ confirms the alkyne derivative **4**. The FTIR spectrum for **5** shows a characteristic N=C=S stretch at 2140 cm^−1^. The ^1^H NMR of **6** shows a cluster of aromatic peaks between *δ*=7.6–7.2 ppm, characteristic for protons of the dibenzylic group.

### ^64^Cu^II^‐labelling experiments

Bispidine ligands form stable complexes very quickly with ^64^Cu^II^ under mild conditions.3a, 3f This was also confirmed for the new bispidines **2**–**6** with additional functional groups in the C9‐position. Quantitative yields (>99 %) of ^64^Cu^II^ complexes with **2**–**6** were obtained within 5 min at room temperature. Radio‐HPLC and radio‐TLC experiments show high radiochemical yield and purity, respectively, with ligand concentrations ≥5 μm at high activity concentrations (100 MBq/200 μL). The molar activity obtained (see the Experimental Section) was higher than 100 MBq nmol^−1^ for bispidines **2**–**6**, whereby the acetic acid‐functionalised bispidine **2** gives the highest value of >500 MBq nmol^−1^ (Table 1). The distribution ratio (log *D*_o/w_) of ^64^Cu^II^‐bispidine complexes was determined in a two‐phase 1‐octanol/buffer system at physiologically relevant pH values (see the Experimental Section). This allows for information about the hydrophilic or lipophilic character of the complexes and thus permits the reliable evaluation of properties such as adsorption, distribution, metabolism, and excretion (ADME) in living systems.^11^ As summarised in Table 1, ^64^Cu^II^ complexes of bispidines **2**–**6** are hydrophilic in character with log *D* values ranging from −4 (^64^Cu^II^‐**2**) to −1.2 (^64^Cu^II^‐**5**).

**Table 1:** Distribution ratios (log *D*_o/w_) at different pH and molar activity *A*_m_ of ^64^Cu^II^‐bispidine complexes.

^64^Cu^II^–bispidine complex	pH			*A*_m_
	7.2	7.4	7.6	[MBq nmol^−1^]
^64^Cu‐**2**	−4.04±0.06	−4.07±0.01	−3.91±0.07	522±10
^64^Cu‐**3**	−2.36±0.08	−2.32±0.07	−2.28±0.09	420±15
^64^Cu‐**4**	−3.27±0.02	−3.43±0.03	−3.21±0.03	222±18
^64^Cu‐**5**	−1.25±0.03	−1.27±0.02	−1.40±0.08	181±12
^64^Cu‐**6**	−1.71±0.09	−1.71±0.01	−1.76±0.07	175±15

The rapid complexation kinetics under mild conditions, high molar activities and appropriate hydrophilic properties of ^64^Cu^II^‐labelled bispidines make these BFCs attractive for further in vitro and in vivo experiments.

### Stability assessment by ligand‐exchange studies

To estimate the stability of the ^64^Cu^II^‐labelled bispidine BFCs **2**–**6**, ligand exchange reactions in the presence of a 1000‐folds excess of the competitive ligands EDTA and DOTA for up to 24 h at 37 °C were studied. The evaluation was conducted using radio‐TLC by which the studied complexes were easily distinguished from [^64^Cu]Cu‐EDTA and [^64^Cu]Cu‐DOTA, as the radiolabelled bispidine derivatives **2**–**6** have a retention factor (*R*_f_) of 0.6–0.7 and ^64^Cu^II^ coordinated to EDTA and DOTA moves with the solvent front (Supporting Information Figure S7). The percent of ^64^Cu^II^ exchanged between ligands is tabulated for 1, 2, 4, and 24 h in the Supporting Information, Table S1. At a 1000‐fold molar excess of EDTA or DOTA, the different bispidine derivatives show no transchelation throughout the period of investigation, confirming their excellent Cu^II^ complex stabilities. The modification of the bispidine backbone at the C9‐position has obviously no influence on the stability and inertness of the ^64^Cu^II^ complexes formed.

### Synthesis and radiolabelling of bispidine–cetuximab conjugate (5′)

**Figure 1:**
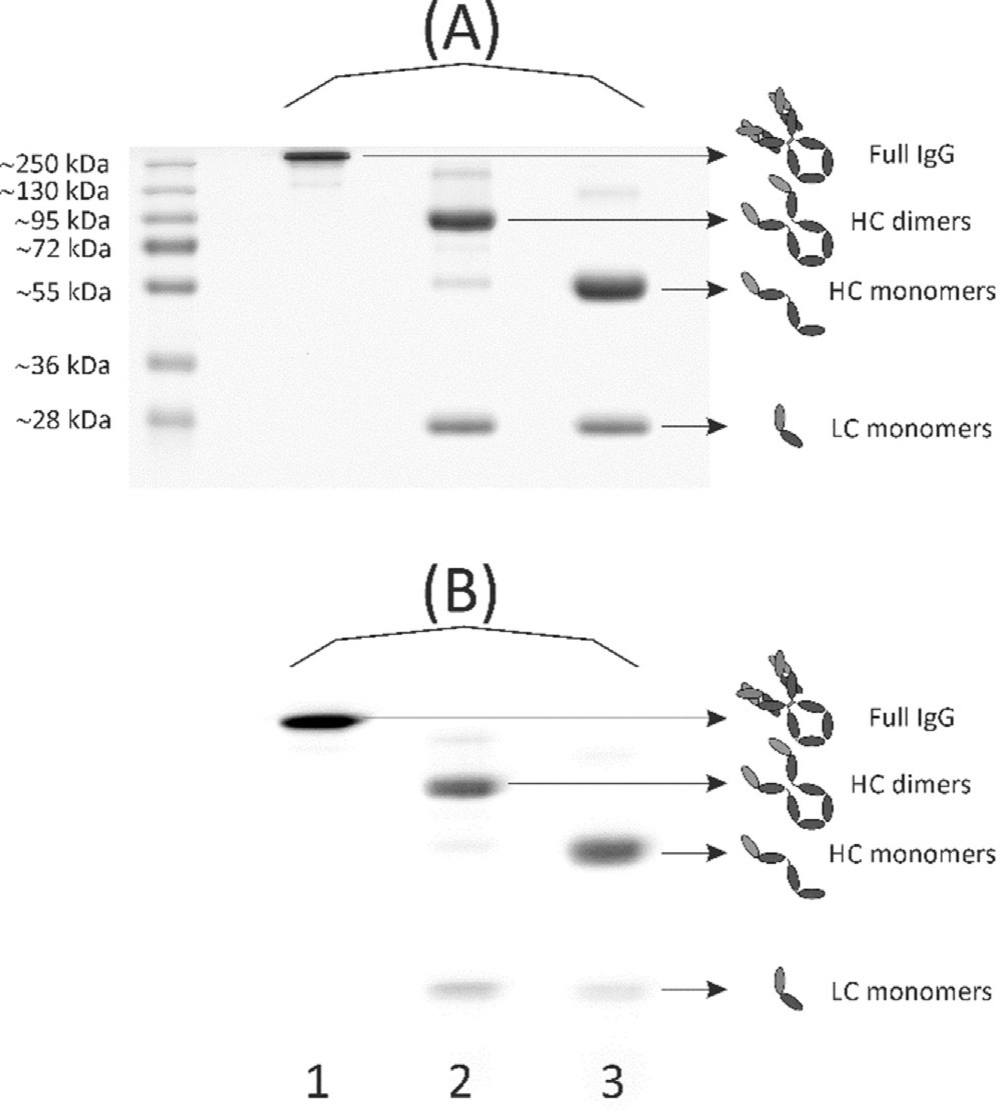
SDS‐PAGE analysis of ^64^Cu^II^‐labelled bispidine‐conjugated cetuximab (**5**′). Coomassie stained 12 % SDS‐polyacrylamide gel (A) and autoradiography (B) showing bands of 5′ corresponding to the intact IgG molecule (≈150 kDa), the monomeric light (LC, ≈25 kDa) and heavy (HC, ≈50 kDa) chain as well as to the dimeric heavy chain (≈100 kDa). Protein samples in lane 1 were not reduced or denatured before electrophoresis. Samples in lane 2 were reduced before gel loading, whereas samples in lane 3 were reduced as well as heat‐denatured prior to electrophoretic separation.

For the classical, non‐specific conjugation using the isothiocyanate‐functionalised bispidine derivative **5**, the well‐studied, FDA‐approved therapeutic monoclonal antibody (mAb) cetuximab (C225; Erbitux, ImClone LLC) served as a model protein.^12^ This chimeric human murine IgG1 molecule binds specifically to the extracellular domain of EGFR, a receptor tyrosine kinase that is often overexpressed in human malignancies.^13^ Cetuximab contains in total 86 lysine residues, eleven in each light and 32 in each heavy chain.^14^ Similar to a previous study,^15^ the mAb was modified with **5** via random isothiocyanate‐mediated coupling. After purification, the degree of conjugation was determined by MALDI‐TOF MS analysis (Supporting Information, Figure S8). Two to three molecules of bispidine were on average conjugated to cetuximab as each chelator subunit adds approximately 843 g mol^−1^ to the molecular weight of the mAb. The bispidine–cetuximab conjugate **5**′ was successfully labelled with ^64^Cu (Supporting Information, Figure S9) and afterwards analysed by gel electrophoresis and autoradiography (Figure 1). The detection of the light and the heavy chains in the autoradiograph prove the attachment of bispidine chelators to both structural elements of the antibody.

These findings illustrate that random bioconjugation to the side‐chain *ϵ*‐amine of lysine residues inevitably leads to a twofold heterogeneity. First, the product population contains conjugates with a variable bispidine‐to‐antibody ratio. Second, conjugates with the same bispidine‐to‐antibody ratio are likely regioisomers as many surface‐accessible lysine residues are potential sites for conjugation with **5**. In other words, in the obtained product population the conjugates do not only differ in the amount of bispidines they are bearing, but also in the sites where the chelators are attached. Consequently, each conjugate of that vastly heterogeneous mixture can have its own set of properties regarding key parameters such as pharmacokinetics, solubility, stability, biological potency, antigen affinity and immunoreactivity.^16^ Regarding the latter, exhaustive modification of lysine residues at or next to the antigen‐binding site may lead to steric hindrance of antigen recognition and interaction.^17^ In particular, for small‐sized antibody formats such as single‐chain variable fragments (scFvs) or single‐domain antibodies (sdAbs), the risk of affecting key amino acids is huge due to their compact structure and limited number of available functional reactive residues outside the antigen‐binding site. These severe drawbacks of random conjugation strategies represent major challenges in the clinical translation and market approval of antibody‐derived conjugates as they impede consistent and reproducible manufacturing as well as straightforward characterisation.

**Scheme 2:**
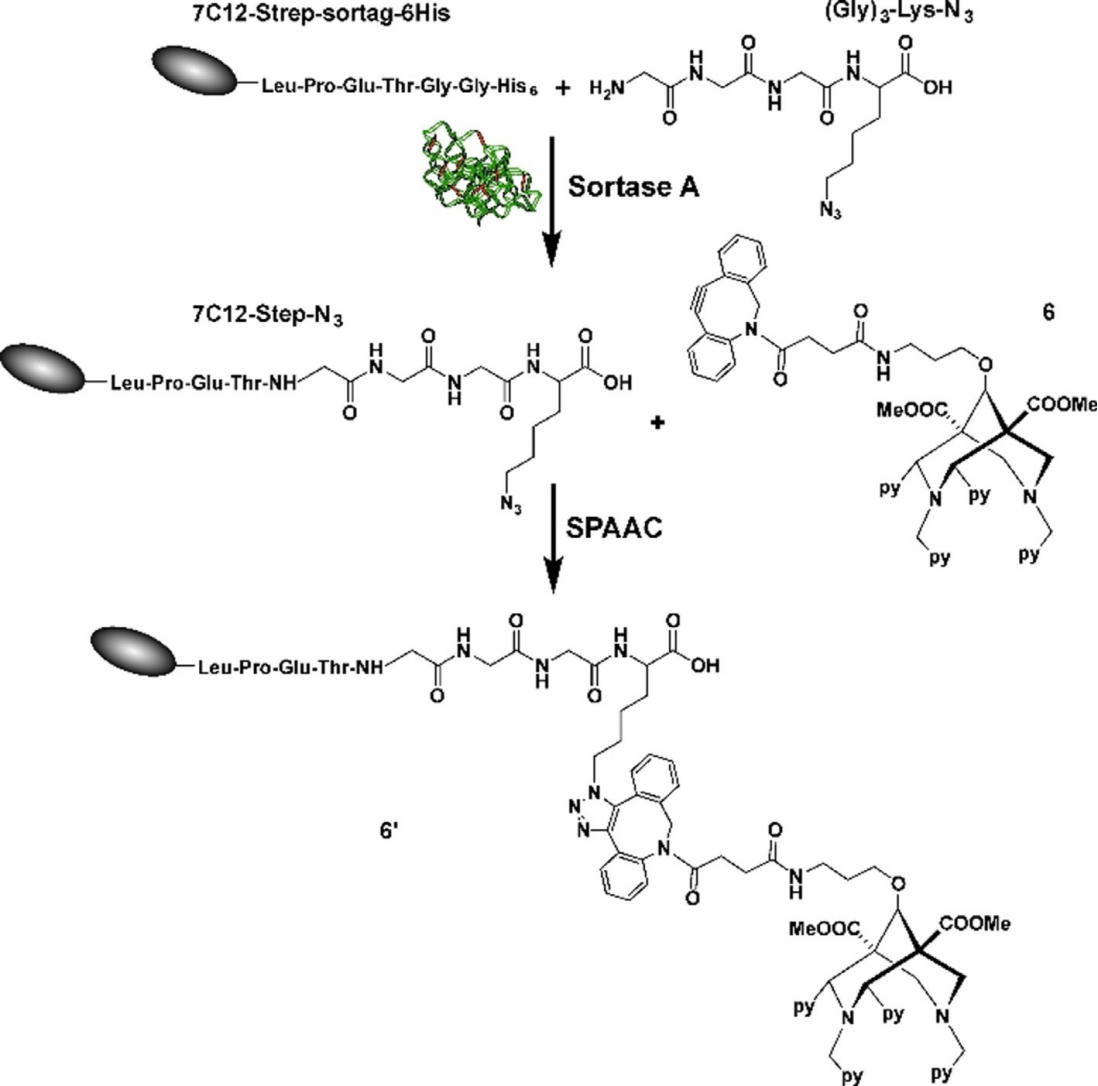
Overview of the two‐step site‐specific antibody modification and conjugation using a combination of enzyme‐mediated bioconjugation and click chemistry. The first step uses enzymatic bioconjugation with the transpeptidase Sortase A for site‐specific incorporation of a functional azide group and the second step involves the azide–alkyne cycloaddition click reaction.

Hence, significant efforts are made to obtain a homogeneous product population by pursuing site‐specific conjugation strategies.16a, 17b, 18 With this in mind, we herein adapted the two‐step site‐specific modification introduced recently, combining chemoenzymatic bioconjugation and click chemistry.^19^ The first step of our approach uses Sortase‐mediated bioconjugation for regioselective incorporation of an azide‐containing linker into an EGFR‐specific sdAb. The second step of this modular approach involves the modification of the azide‐tagged antibody fragment with the DBCO‐modified bispidine derivative **6** via Cu‐free strain‐promoted alkyne–azide cycloaddition (Scheme 2).

### Synthesis and radiolabelling of the bispidine‐sdAb conjugate (6′)

#### Sortase A‐mediated azide functionalisation

The efficiency of chemoenzymatic functionalisation was evaluated using the EGFR‐specific sdAb 7C12 and (Gly)_3_‐Lys‐N_3_ as a substrate. The sdAb was produced with a C‐terminal (GGGGS)_3_ spacer, followed by a Strep‐tag, the LPETGG sortase motif, another (GGGGS)_3_ spacer and a hexahistidine purification tag, resulting in 7C12‐Strep‐sortag‐6His. A successful Sortase‐mediated conjugation leads to the elimination of the hexahistidine tag. This design allows the removal of the unreacted sdAb and the isolation of the pure 6His‐tagged enzyme by IMAC.^20^ To optimise the reaction, the molar ratios of SrtA, sdAb and (Gly)_3_‐Lys‐N_3_ were varied (Supporting Information Figure S10) and a ratio of 1:1:10 was identified to be optimal.

#### Strain‐promoted azide–alkyne cycloaddition

**Figure 2:**
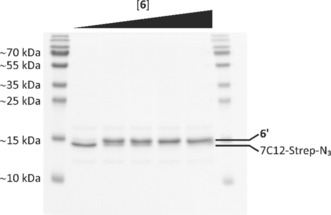
SDS‐PAGE analysis of strain‐promoted azide‐alkyne cycloaddition between **6** and azide‐functionalised single‐domain antibody 7C12‐Step‐N_3_. The molar amount of **6** was varied (1–10 nmol), while the amount of the azide‐modified sdAb was kept constant (1 nmol).

The site‐specifically azide‐modified sdAb 7C12‐Strep‐N_3_ was reacted with **6** for up to 4 h at 25 °C. To optimise the reaction, the molar rations of sdAb and **6** were varied (Figure 2).

**Figure 3:**
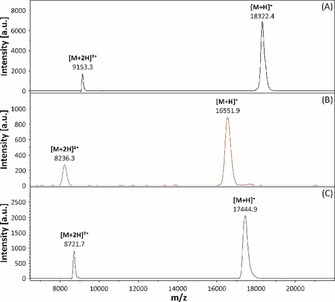
MALDI‐TOF mass spectra of the purified sdAb derivative 7C12‐Strep‐sortag‐6His (A), the azide‐functionalised intermediate 7C12‐Strep‐N_3_ (B) and the single‐conjugated **6′** (C).

A reaction with a molar ratio sdAb:**6** of 1:5 was identified as being ideal. After purification of the reaction mixture by size exclusion chromatography, the obtained conjugate was analysed by MALDI‐TOF MS (Figure 3). The mass spectra of the final purified product showed a homogeneous population of a single‐conjugated **6**′ with a molecular mass of ≈17.4 kDa (Table S2, Supporting Information).

#### Radiolabelling of bispidine‐modified sdAb 6′

**Figure 4:**
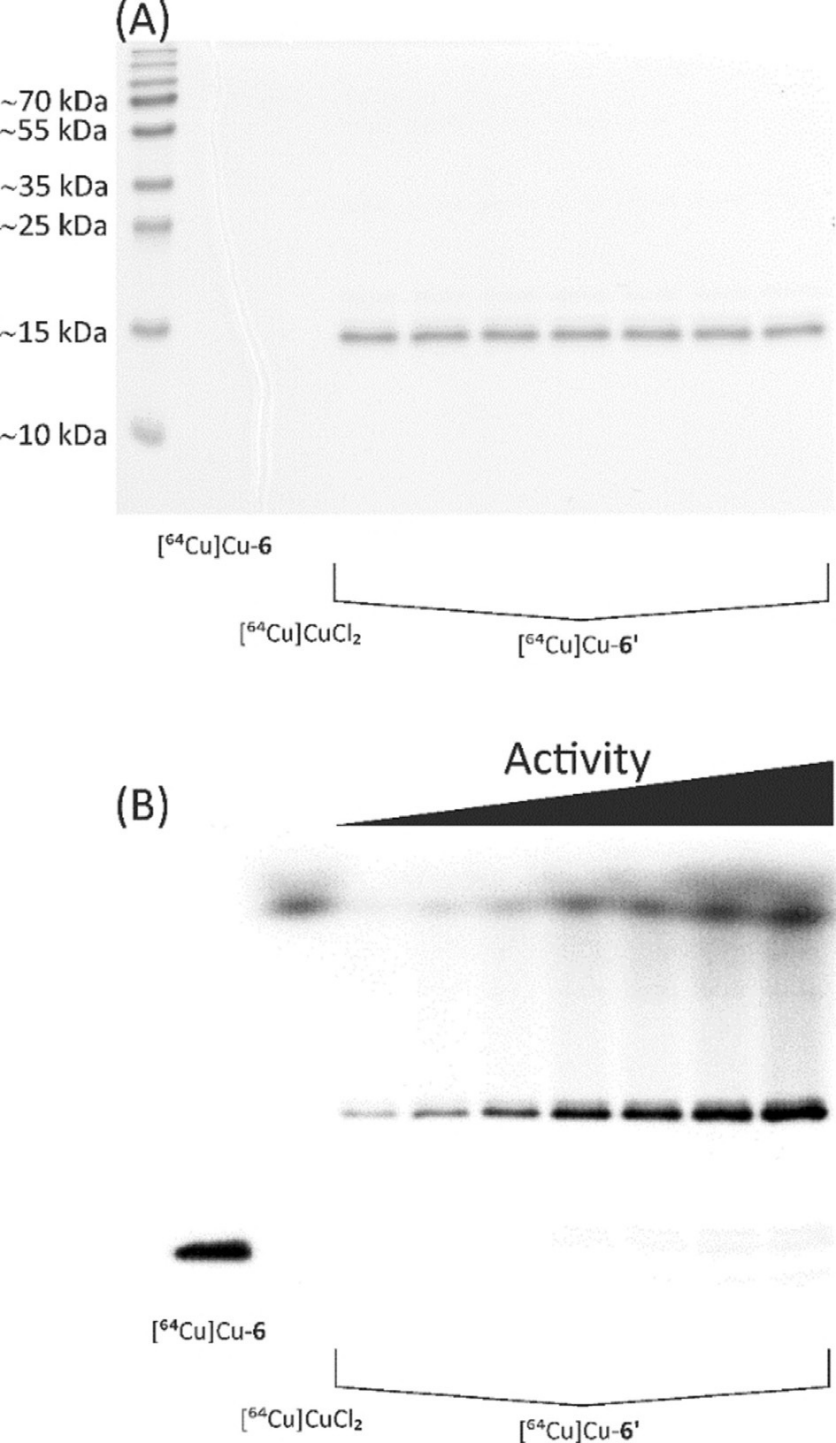
Discontinuous SDS‐PAGE and autoradiography analysis of [^64^Cu]Cu^II^‐**6**′ before purification. After radiolabelling, aliquots of each reaction were separated using a 15 % SDS‐polyacrylamide gel, and prior to staining with Coomassie staining (A), an electronic autoradiographic image of the gel was recorded using a radioluminography laser scanner (B).

Bispidine‐sdAb **6**′ (1 nmol) was radiolabelled with [^64^Cu]CuCl_2_ with activities ranging from 0.5 to 10 MBq, and the extent of radiometal complexation was assessed by radio‐TLC (Supporting Information, Figure S11). As expected, the amount of radiolabelled protein correlated with the activity of ^64^Cu^II^ added. In addition to radio‐TLC, ^64^Cu^II^‐labelling of **6**′ was confirmed by SDS‐PAGE and subsequent autoradiography. A single predominant protein species corresponding to the mononuclear ^64^Cu^II^‐labelled sdAb is clearly visible in the Coomassie stained gels as well as in the autoradiographic images (Figure 4). Free ^64^CuCl_2_ is detectable at the upper part of the gel, whereas the reference [^64^Cu]Cu^II^‐**6** migrates with the gel front. The reaction mixture was subsequently purified by spin filtration to completely remove free ^64^CuCl_2_ until ^64^Cu^II^‐**6**′ was finally obtained with a radiochemical yield of ≈99 %.

### In vitro characterisation of single‐conjugated sdAbs

**Figure 5:**
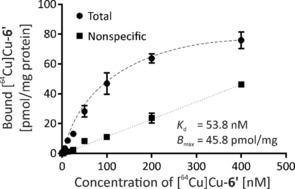
Analysis of binding affinity of [^64^Cu]Cu^II^‐**6**′ to human EGFR‐presenting cells. Total binding was measured in the absence of, and nonspecific binding in the presence of 4 μm unlabelled sdAb (7C12‐Strep‐sortag‐6HIS).

The functionality of the functionalised **6**′ after the two‐step site‐specific modification was evaluated by cell binding studies using the human epithelial cell line A431 originating from an epidermoid carcinoma of the skin. These squamous carcinoma cells express approximately 2×10^6^ EGFR molecules per cell, which represents a high expression level.^21^ In a saturation binding assay, in which the amount of radioligand required to saturate these receptors is measured, the equilibrium dissociation constant *K*_d_ and the total number of receptors expressed on the cell surface *B*_max_ were determined (Figure 5).

Nonlinear regression analysis of the saturation curve data revealed a *K*_d_ value of 53.8±8.6 nm and a *B*_max_ of 45.8±2.7 pmol mg^−1^ protein. The apparent affinity determined is in accordance with previously obtained data for ^99m^Tc‐labelled 7C12.^22^ The bispidine conjugation via the two‐step site‐specific modification procedure does obviously not affect the binding properties of the sdAb.

**Figure 6:**
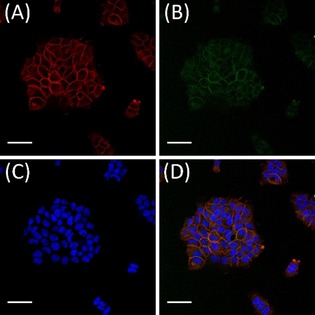
Co‐localisation of **6**′ and EGFR on human epidermoid carcinoma cells. A431 cells were incubated with 100 nm **6**′ for 4 h at 37 °C. Binding of the immunoconjugates to plasma membrane localised EGFR was analysed by indirect immunofluorescence. To confirm the expression of EGFR, an anti‐EGFR Alexa Fluor 647 antibody conjugate was used (A; red fluorescence). An anti‐Strep‐tag Chromeo 488 conjugate was used to detect the Strep‐tagged 6′ (B; green fluorescence). The nuclei were visualised by the DNA binding stain Hoechst 33258 (C; blue fluorescence). The overlay of (A), (B) and (C) is shown in (D). Scale bars=20 μm.

To investigate the binding and localisation of these immunoconjugates at the cellular level, fluorescence microscopic imaging was performed (Figure 6). Confocal imaging of A431 cells showed binding of the bispidine‐functionalised sdAbs to the plasma membrane of these human epidermoid carcinoma cells and co‐localisation with EGFR indicating the preserved targeting ability of 7C12 after site‐specific modification. As observed earlier, **6**′ showed a predominant membrane staining even after 24 h of incubation, and only very little intracellular fluorescence was observed (Supporting Information, Figure S12). These observations are in agreement with former studies,^22^, ^23^ indicating that bispidine conjugation has no detrimental effect on the EGFR binding behaviour or on the specificity of the sdAb.

### PET imaging of single‐conjugated sdAbs in A431 tumour xenografts

**Figure 7:**
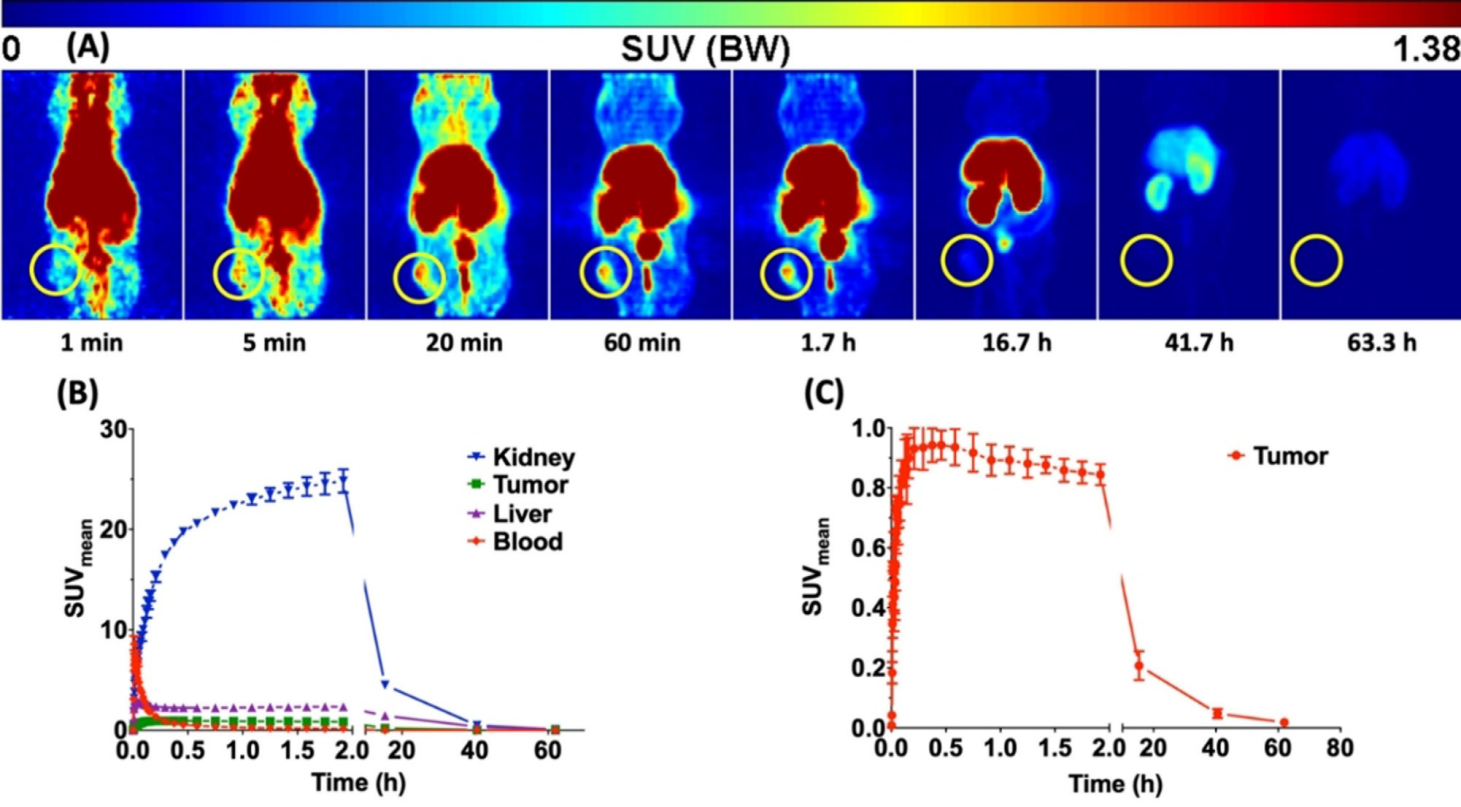
PET dynamic imaging. Maximum Intensity Projections (MIP) of a PET study with ^64^Cu^II^‐labelled 6′ in an A431 tumour mouse after single intravenous injection into the tail vein. The tumour is localised inside the yellow circle (A). Representative time‐activity curves of kidney, tumour, liver and blood (B). Time‐activity curve of the tumour (C). Data are shown as mean ± SEM of two animals.

The promising in vitro results strongly encouraged us to investigate the in vivo distribution of this ^64^Cu^II^‐labelled bispidine‐sdAb conjugate **6**′ in a tumour mouse. Tumour xenograft (epidermoid carcinoma) was obtained after subcutaneous injection of A431 cells into the right thigh of a female NMRI nu/nu mouse. A dynamic PET imaging study was performed when the tumour was about 10 mm. Conjugate **6**′ was labelled with [^64^Cu]CuCl_2_ for 60 min at room temperature, resulting in a radiochemical yield of 91.9 % and molar activity of 27.7 GBq μmol^−1^ without further purification. 39 MBq (2 nmol ^64^Cu^II^‐**6**′) were injected into a tail vein of A431 tumour bearing mice (*n*=2). As shown in Figure 7, the ^64^Cu^II^‐labelled bispidine‐sdAb conjugate **6′** was rapidly cleared via the renal pathway with a blood activity half‐life of 28.2 min, which is expected due to the hydrophilic character and the relatively low molecular weight for a protein. Liver uptake was likely elicited by free ^64^Cu^II^ in the injection solution. The tumour is clearly visible after 5 min and the activity peak was reached after 22.5 min. After one hour, most of the activity was removed from the blood and the tumour was sharply delimited, leading to a tumour‐to‐muscle ratio above 13. The tumour is visible with high contrast between 20 min and 2 h. Overall, the pharmacokinetic properties of ^64^Cu^II^‐**6**′ are as expected and similar to the data obtained for a ^99m^Tc‐labelled sdAb 7C12.^24^

## Conclusion

Bispidines with their excellent metal chelation properties and promising bioconjugation strategies are outstanding bifunctional imaging agents. Due to their high ^64^Cu^II^ selectivity, ease of biofunctionalisation and high in vivo stability, the hexadentate bispidine derivatives reveals an enormous potential as PET imaging agent for cancer diagnosis. The C9‐modified bispidines (**2**–**6**) that can be readily coupled to cancer targeting moieties have been synthesised and characterised. The DBCO‐functionalised ligand **6** was designed to undergo strain‐promoted azide–alkyne coupling to single‐domain antibodies, and the corresponding site‐specifically single‐conjugated sdAb derivative with uncompromised functionality was obtained by a combination of enzyme‐mediated bioconjugation and click chemistry. The introduction of the azide handle at the C‐terminal end of the sdAb makes this strategy a generic approach for a wide range of dibenzocyclooctyne‐containing substrates. As the herein targeted antigen EGFR is present in practically all squamous cell and some other cancers occurring in the clinic, the bispidine‐based selective and highly specific antibody fragments labelled with ^64^Cu^II^ hold a promise to deliver important therapy planning and monitoring options for head and neck, oesophageal, gastric, bladder and other mucosal cancer patients.

## Experimental Section

### General

All chemicals were used without further purification and in the highest degree of purity. ^1^H and ^13^C NMR spectra were recorded with a Varian Inova‐400 spectrometer. The chemical shifts are reported relative to the standard TMS. IR spectra were measured with a Fisher Scientific Nicolet iS5 FTIR spectrometer. For mass spectrometry, a QuadroLC by Micromass with electrospray ionisation (ESI) mode and a Bruker MALDI‐TOF MS instrument were used. The following HPLC‐system was used for analytical runs: Knauer‐Smartline including a pump 1000, a manager 5000 and software Chromgate 2.8 using an Aqua C‐18 (Phenomenex) column 125 Å (250×4.6 mm, 5 μm). The same equipment was used for radioactive labelling studies with the radioactivity detector Raytest Ramona Star. The purification was performed by HPLC system: Knauer Smartline, including a pump 1000, a UV detector 2500, and a manager 5000 using semi‐preparative HPLC columns: Jupiter Proteo (Phenomenex) 90 Å (250×10 mm, 4 μm) for compounds **2** and **4**, and preparative HPLC column Gemini C‐18 (Phenomenex) 110 Å (250×10 mm, 5 μm) for compounds **3**, **5**, and **6**. HPLC was performed using the two eluents (eluent A: acetonitrile, containing 0.1 % trifluoroacetic acid; eluent B: water, containing 0.1 % trifluoroacetic acid). TLC was performed using neutral Al_2_O_3_ plates (Polygram), developed in a 1:1 mixture of 2 m NH_4_OAc (pH 6.0) and MeOH (^64^CuCl_2_, *R*_f_=0; ^64^Cu‐bispidine, *R*_f_=0.7) and analysed with a Raytest Linear analyser RITA. UV/Vis spectra were recorded using an Analytik Jena SPECORD 50. The elemental analysis was performed on a Leco Elemental Analyser CHNS‐932.

### Dimethyl‐9‐hydroxy‐2,4‐dipyridin‐2‐yl‐3,7‐bis(pyridin‐2‐ylmethyl)‐3,7 diazabicyclo[3.3.1] nonane‐1,5‐dicarboxylate (1)

The bispidine ligand **1** was prepared according the published procedure.3f, 25

### 2‐{[‐1,5‐bis(methoxycarbonyl)‐2,4‐di(pyridin‐2‐yl)‐3,7‐bis(pyridin‐2‐ylmethyl)‐3,7‐diazabicyclo[3.3.1]nonan‐9‐yl]oxy}acetic acid (2)

To a solution of **1** (50 mg, 84.2 μmol) in 10 mL of THF, NaH (10 mg, 416 μmol) was added. The solution was stirred for 30 min at 50 °C. Afterwards, iodoacetic acid (17.65 mg) was added to the solution and the mixture was stirred for 2 h under reflux. The reaction was then quenched by slow addition of distilled water (5 mL) and the solvent was removed reduced under pressure. The crude product was purified by HPLC and obtained as a white solid. Yield: 4.3 mg (6.6 μmol), 7.8 %; HPLC: 30 % to 70 % A in 32 min, flow rate=10 mL min^−1^, retention time (*t*_R_)=12.93 min; ESI MS (*m*/*z*): [*M*+H]^+^ 653.1 (calcd for C_35_H_36_N_6_O_7_^+^: 653.26); ^1^H NMR: (400 MHz, CD_3_CN) *δ*=9.01 (d, 1 H), 8.64 (d, *J=*5.4 Hz, 1 H), 8.18–8.14 (m, 2 H), 8.02 (td, *J=*7.7, 1.8 Hz, 2 H), 7.83–7.79 (m, 1 H), 7.75–7.70 (m, 2 H), 7.68–7.65 (m, 2 H), 7.47 (t, *J=*6.7 Hz, 1 H), 7.41 (d, *J=*7.8 Hz), 7.24 (ddd, *J=*7.7, 4.8, 1.1 Hz, 2 H), 6.90 (d, *J=*7.9 Hz, 1 H), 5.14 (d, *J=*1.4 Hz, 1 H), 5.06 (s, 1 H), 4.65 (d, *J=*2.1 Hz, 4 H), 4.58 (s, 2 H), 3.94 (d, *J=*12.6 Hz, 2 H), 3.72 (d, *J=*12.7 Hz, 2 H), 3.65 (s, 6 H); FTIR: 3062 (O‐H, C−H), 1737 (C=O), 1667 (C=N), 1181 (C−H), 1129 ppm (C−H).

### *tert*‐Butyl (3‐bromopropyl)carbamate

To a solution of 3‐bromopropylamine hydrobromide (2.0 g, 9.1 mmol) in DCM (30 mL), TEA (5 mL, 36.5 mmol) was added and the reaction solution was stirred at room temperature for 30 min before di‐*tert*‐butyl‐dicarbonate (2.4 g, 10.9 mmol) was added. After 12 h of continuous stirring under a nitrogen atmosphere, 20 mL ethyl acetate and 20 mL water were added to the solution. The organic phase was separated, washed with brine and the solvent was removed under reduced pressure. The product was obtained as a white solid by after purification by column chromatography (silica gel, ethyl acetate:hexane=1:5). Yield: 1.52 g, 70 %; ^1^H NMR: (400 MHz, CDCl_3_) *δ*=3.37 (t, *J*=6.5 Hz, 2 H), 3.19 (q, *J*=4.7, 4.0 Hz, 2 H), 1.97 (p, *J*=6.6 Hz, 2 H), 1.37 ppm (s, 9 H); ^13^C NMR: *δ*=155.99, 66.80, 39.69, 30.74, 28.71–27.90 ppm (m).

### Dimethyl‐9‐{3‐[(*tert*‐butoxycarbonyl)amino]propoxy}‐2,4‐di(pyridin‐2‐yl)‐3,7‐bis(pyridin‐2‐ylmethyl)‐3,7‐diazabicyclo[3.3.1]nonane‐1,5‐dicarboxylate (3′)

Sodium hydride (311.2 mg, 1.3 mmol) was added to a solution of **1** (300 mg, 504 μmol) in dry THF (50 mL), and the resulting mixture was stirred for 1.5 h at 50 °C. *tert*‐Butyl(3‐bromopropyl)carbamate (238.1 mg, 1.0 mmol) was then added to the solution and stirred for another 20 h at 50 °C. The reaction was quenched by adding saturated sodium hydrogen carbonate solution (50 mL, pH 8). The aqueous layer was extracted with DCM and ethyl acetate. The organic fractions were combined, washed with brine and dried over sodium sulfate. Finally, the organic solvent was removed to obtain crude **3′**. The product was used for the next step in situ. Yield: 131 mg (174 μmol), 35 %; ESI MS (*m*/*z*): [*M*+H]^+^ 751.8 (calcd for C_41_H_50_N_7_O_7_^+^: 752.37); ^1^H NMR: (400 MHz, CDCl_3_) *δ*=8.66 (d, *J=*4.9 Hz, 2 H), 8.44 (dd, *J=*5.0, 1.7 Hz, 2 H), 8.41–8.35 (m, 4 H), 7.89 (d, *J=*7.9 Hz, 3 H), 7.68 (td, *J=*7.6, 1.8 Hz, 2 H), 7.56–7.45 (m, 2 H), 7.37 (dtd, *J=*9.8, 7.6, 1.8 Hz, 8 H), 7.28–7.21 (m, 3 H), 6.99 (ddd, *J=*15.3, 7.5, 5.0 Hz, 7 H), 6.68 (d, *J=*7.7 Hz, 2 H), 5.26 (s, 2 H), 4.90 (d, *J=*6.6 Hz, 2 H), 4.80 (s, 3 H), 4.70 (s, 1 H), 4.36 (s, 2 H), 3.70 (dq, *J=*6.5, 3.3, 2.9 Hz, 2 H), 3.58 (s, 9 H), 3.55–3.45 (m, 9 H), 3.41 (dd, *J=*14.6, 8.1 Hz, 8 H), 3.23 (q, *J=*6.3 Hz, 5 H), 2.99 (q, *J=*6.6, 6.0 Hz, 4 H), 2.55–2.43 (m, 7 H), 2.42 (s, 1 H), 2.06–1.87 (m, 6 H), 1.51 (h, *J=*6.3, 5.5 Hz, 4 H), 1.24–1.20 (m, 3 H), 0.86–0.77 ppm (m, 3 H).

### Dimethyl‐9‐(3‐aminopropoxy)‐2,4‐di(pyridin‐2‐yl)‐3,7‐bis(pyridin‐2‐ylmethyl)‐3,7 diazabicyclo[3.3.1]nonane‐1,5‐dicarboxylate (3)

The Boc‐protected amine (100 mg, 133 μmol) was dissolved in DCM (3 mL), to which trifluoroacetic acid (3 mL) was added. The resulting reaction mixture was stirred for 24 h at room temperature. After addition of chloroform (10 mL), the solvent was removed under reduced pressure. The product **3** was purified by HPLC and obtained as yellow‐brown oil. Yield: 86.5 mg (133 μmol), 100 %; HPLC: 30 % to 70 % A in 15 min, flow rate=10 mL min^−1^, *t*_R_=11 min; ESI MS (*m*/*z*): [*M*+H]^+^ 652.1 (calculated for C_36_H_42_N_7_O_5_^+^: 652.32); ^1^H NMR: (400 MHz, CD_3_CN) *δ*=9.15 (d, *J=*4.9 Hz, 1 H), 8.80 (d, *J=*5.9 Hz, 1 H), 8.14–8.07 (m, 3 H), 8.07–8.00 (m, 1 H), 7.84–7.68 (m, 4 H), 7.64 (d, *J=*7.7 Hz, 1 H), 7.35 (t, *J=*11.8 Hz, 4 H), 7.25 (ddd, *J=*7.8, 4.9, 1.1 Hz, 2 H), 7.19 (d, *J=*8.1 Hz, 1 H), 5.3–5.5 (m, 1 H), 4.95 (d, *J=*1.4 Hz, 2 H), 4.79 (s, 1 H), 4.68 (s, 2 H), 3.81 (t, *J=*6.5 Hz, 3 H), 3.66 (s, 6 H), 2.27–2.08 (m, 1 H), 1.97–1.78 ppm (m, 3 H); ^13^C NMR: (101 MHz, CD_3_CN) *δ*=167.45, 125.12–150.90, 125.33, 80.74, 72.64, 65.12, 55.01, 53.20, 52.14 ppm; FTIR: $\tilde{\nu }$ =3000 (N−H, C−H), 1727 (C=O), 1670 (C=N), 1200 (C−O), 1126 cm^−1^ (N−H).

### Dimethyl‐9‐(prop‐2‐yn‐1‐yloxy)‐2,4‐di(pyridin‐2‐yl)‐3,7‐bis(pyridin‐2‐ylmethyl)‐3,7‐diazabicyclo[3.3.1]nonane‐1,5‐dicarboxylate (4)

Compound **1** (50 mg, 84.17 μmol) was dissolved in dry THF (10 mL) to which sodium hydride (5 mg, 208 μmol) was added. The solution was stirred for 30 min at 50 °C. Propargyl bromide (8.3 μL, 0.1 mmol) was then added to the reaction solution, which was further stirred for 3 h at 50 °C under argon atmosphere. The reaction was quenched by adding a saturated solution of hydrogen carbonate and then extracted with DCM. The organic layer was removed under pressure. The crude product was purified by HPLC as a clear oil. Yield: ≈8 mg (12.6 μmol), 15 %; HPLC: 30 to 70 % A in 40 min, flow rate=10 mL min^−1^, *t*_R_=12.9 min; ESI MS (*m*/*z*): [*M*+H]^+^ 633.4 (calcd for C_36_H_37_N_6_O_5_^+^: 633.28); ^1^H NMR: (400 MHz, CD_3_CN) *δ*=8.90 (d, *J=*4.6 Hz, 1 H, Ar‐H), 8.44 (s, 1 H, Ar‐H), 8.17 (d, *J=*4.5 Hz, 2 H, Ar‐H), 8.00 (td, *J=*7.7, 1.8 Hz, 1 H, Ar‐H), 7.71–7.60 (m, 5 H, Ar‐H), 7.33–7.25 (m, 1 H, Ar‐H), 7.22 (ddd, *J=*7.7, 4.8, 1.1 Hz, 2 H, Ar‐H), 7.15 (d, *J=*7.8 Hz, 2 H, Ar‐H), 6.9 (m, 1 H), 4.99 (d, *J=*1.6 Hz, 2 H, *N*‐CH), 4.76 (s, 1 H, O‐CH), 4.53 (s, 2 H, *N*‐CH_2_), 4.24 (d, *J=*2.4 Hz, 2 H, O‐CH_2_), 4.15 (d, *J=*12.6 Hz, 2 H, *N*‐CH_2_), 3.58 (s, 6 H, O‐CH_3_), 3.53 (s, 1, *N*‐C‐H_2_), 3.56 (d, *J=*1.6 Hz, 1 H, C‐H_2_), 3.50 (s, 2 H,*N*‐C‐), *δ*=2.82 ppm (t, *J=*2.4 Hz, 1 H); FTIR: $\tilde{\nu }$ =2359 (C≡C), 1731 (C=O), 1687 (C=N), 1180 (C−H), 1131 cm^−1^ (C−H).

### Dimethyl‐9‐{3‐[3‐(4‐isothiocyanatophenyl)thioureido]propoxy}‐2,4‐di(pyridin‐2‐yl)‐3,7‐bis(pyridin‐2‐ylmethyl)‐3,7‐diazabicyclo[3.3.1]nonane‐1,5‐dicarboxylate (5)

Compound **3** (250 mg, 384 μmol) was dissolved in DCM (25 mL). *p*‐phenylene diisothiocyanate (80 mg, 416 μmol) and TEA (1 mL, 7.1 mmol) were added to this solution. The reaction mixture turned yellow in 5–10 min. The reaction was stirred at room temperature for 16 h. Solvent was evaporated under reduced pressure. Unreacted *p*‐phenylene diisothiocyanate was extracted from the mixture by dietyl ether. The crude solid **5** was then purified by RP‐HPLC. Yield: 194 mg (230 μmol), 60 %; HPLC: 30 % to 70 % A in 15 min followed by 70 to 100 % in 15 min, flow rate=10 mL min^−1^, *t*_R_=19 min; ESI MS (*m*/*z*): [*M*+H]^+^ 844.3 (calculated for C_44_H_46_N_9_O_5_S_2_^+^: 844.3); ^1^H NMR: (400 MHz, CDCl_3_) *δ*=8.90 (ddd, *J=*4.8, 1.8, 0.9 Hz, 1 H), 8.63 (d, *J=*8.1 Hz, 1 H), 8.23–8.12 (m, 2 H), 7.93 (ddd, *J=*4.9, 1.8, 0.8 Hz, 2 H), 7.82 (td, *J=*7.7, 1.8 Hz, 1 H), 7.71–7.53 (m, 5 H), 7.45 (ddd, *J=*7.4, 5.8, 1.2 Hz, 2 H), 7.27–7.17 (m, 7 H), 7.15–7.05 (m, 4 H), 4.69–4.60 (m, 5 H), 4.01 (d, *J=*12.5 Hz, 2 H), 3.92 (d, *J=*12.7 Hz, 2 H), 3.80 (s, 2 H), 3.66 (t, *J=*5.3 Hz, 4 H), 3.53 (s, 6 H), 1.84 ppm (p, *J=*5.6 Hz, 2 H); ^13^C NMR: (101 MHz, CDCl_3_) *δ*=182.00, 149.34, 138.00, 126.02, 125.76, 123.87, 28.60 ppm; FTIR: $\tilde{\nu }$ =>3000 (N−H, C−H), 2140 (N=C=S), 1727–1675 (C=O), 1200 (C−O), 1140 cm^−1^ (N−H); EA: C: 53.75 %, N: 11.14 %, H: 4.61 %, S: 5.46 %.

### Dimethyl‐9‐[5,8(8‐dibenzocyclooctyne)diamido)octoxy]‐2,4‐di(pyridin‐2‐yl)‐3,7‐bis(pyridin‐2‐ylmethyl)‐3,7‐diazabicyclo[3.3.1]nonane‐1,5‐dicarboxylate (6)

Compound **3** (2.5 g, 3.84 mmol) and TEA (2.5 mL, 17.8 mmol) were dissolved in DCM (40 mL). DBCO‐NHS ester (1.0 g, 2.5 mmol, Jena Bioscience GmbH) dissolved in 10 mL DCM was added to this solution. The reaction mixture was stirred for 2 h at room temperature. The product **6** was concentrated under reduced pressure, and purified by HPLC analysis. Yield: 1.63 g (1.73 mmol), 45 %; HPLC: 30 to 70 % A in 15 min, flow rate=10 mL min^−1^, *t*_R_=22 min; ESI MS (*m*/*z*): [*M*+H]^+^ 939.1 (calcd for C_55_H_54_N_8_O_7_^+^: 939.41); ^1^H NMR: (400 MHz, CDCl_3_) *δ*=8.89–8.82 (m, 1 H), 8.49 (s, 1 H), 8.26 (s, 1 H), 8.23 (d, *J=*8.1 Hz, 1 H), 8.11 (d, *J=*5.2 Hz, 2 H), 8.02 (d, *J=*8.9 Hz, 2 H), 7.97–7.82 (m, 2 H), 7.70–7.60 (m, 4 H), 7.60–7.47 (m, 4 H), 7.47–7.40 (m, 2 H), 7.40–7.32 (m, 5 H), 7.29 (d, *J=*8.1 Hz, 6 H), 7.14–7.04 (m, 3 H), 5.14 (s, 1 H), 5.12–5.02 (m, 1 H), 5.00 (s, 1 H), 4.73–4.64 (m, 2 H), 4.62 (s, 1 H), 4.07 (d, *J=*9.6 Hz, 3 H), 4.00 (d, *J=*13.3 Hz, 2 H), 3.90 (d, *J=*10.4 Hz, 3 H), 3.68–3.59 (m, 3 H), 3.50 (t, *J=*4.1 Hz, 8 H), 3.27 (d, *J=*9.1 Hz, 1 H), 3.08 (t, *J=*6.0 Hz, 3 H), 2.80–2.66 (m, 8 H), 2.46 (dt, *J=*14.6, 7.3 Hz, 5 H), 2.29 (ddt, *J=*21.6, 14.4, 7.1 Hz, 4 H), 2.15 (s, 1 H), 1.95 (dt, *J=*16.6, 7.1 Hz, 2 H), 1.58 (s, 1 H), 1.52 (s, 1 H), 1.24 (s, 2 H), 0.82 ppm (s, 1 H); FTIR: >3000 (N−H, C−H), 1740–1600 (C=O), 1200 (C−O), 1140 (N−H).

### *Escherichia (E.) coli* strains and plasmids

*E. coli* NEB 5‐alpha (fhuA2 Δ(argF‐lacZ)U169 phoA glnV44 Φ80Δ (lacZ)M15 gyrA96 recA1 relA1 endA1 thi‐1 hsdR17) was used in molecular cloning experiments, whereas *E. coli* SHuffle T7 Express (fhuA2 lacZ:T7 gene1 [lon] ompT ahpC gal λatt:pNEB3‐r1‐cDsbC (Spec^R^, lacI^q^) ΔtrxB sulA11 R(mcr‐73:miniTn10–Tet^S^)2 [dcm] R(zgb‐210:Tn10–Tet^S^) endA1 Δgor Δ(mcrC‐mrr)114:IS10) and *E. coli* BL21(DE3) (fhuA2 [lon] ompT gal (*λ* DE3) [dcm] ΔhsdS) were used for expression of the recombinant proteins. All strains were purchase from New England Biolabs. The generation of pET‐28b:7C12 encoding the EGFR‐specific single‐domain antibody 7C12 has been described previously.^22^ The plasmid pGBMCS‐SortA was a gift from Fuyuhiko Inagaki (Addgene plasmid #21931).^26^

### Molecular cloning

A DNA fragment coding for a (GGGGS)_3_ spacer followed by a Strep‐tag, the LPETGG sortase motif and another (GGGGS)_3_ spacer was synthesised including a 5′ restriction site for HindIII and a 3′ restriction site for *Xho*I, respectively. The ≈150 nt fragment was digested with appropriate restriction endonucleases and ligated in‐frame into HindIII/*Xho*I‐linearised pET‐28b:7C12 plasmid.^22^ The ligation reactions were transformed into chemically competent *E. coli* NEB 5‐alpha cells. The DNA sequences of the resulting recombinant construct pET‐28b:7C12‐Strep‐sortag‐6HIS were checked by Sanger sequencing.

### Cultivation and expression of recombinant proteins

Freshly transformed *E. coli* SHuffle T7 Express or *E. coli* BL21(DE3) harbouring the plasmids pET‐28b:7C12‐Strep‐sortag‐6HIS or pGBMCS‐SortA were inoculated in 10 mL of LB broth containing 50 μg mL^−1^ of kanamycin or 100 μg mL^−1^ of ampicillin, respectively, and cultivated at 30 °C overnight in an orbital shaker with 50 mm offset and shaking speed of 200 rpm. After that, 5 mL of this pre‐culture were transferred into 100 mL MagicMedia *E. coli* Expression Medium (Life Technologies) in 500 mL baffled‐bottom glass flasks and grown at 30 °C for 24 h. For final harvest, cultures were chilled on ice for 5 min and centrifuged for at least 15 min at 6000×g and 4 °C. After removal of the supernatant, cell pellets were either stored at −20 °C or subjected to the purification procedure immediately.

### Purification of recombinant proteins

A high‐capacity Ni‐iminodiacetic acid (IDA) resin in combination with an ÄKTA pure chromatography system (GE Healthcare) was used for purification of hexahistidine tagged proteins by immobilised metal affinity chromatography (IMAC) under native conditions.

Efficient cell lysis was achieved by addition of 1 mL RIPA cell lysis buffer (G‐Biosciences) supplemented with EDTA‐free protease and phosphatase inhibitor cocktail (Thermo Fisher Scientific), 500 μg lysozyme (Sigma–Aldrich) and 25 U endonuclease (Thermo Scientific Pierce) per 200 mg bacterial cell pellet. Prior to incubation on ice for at least 15 min, the pelleted cells were resuspended completely by vortexing or pipetting up and down until no cell clumps remained. After centrifugation at 10 000×g and 4 °C for 20 min to remove cellular debris, the clarified supernatant was loaded using an automated sample pump with a flow rate of 0.5 mL min^−1^. IMAC was performed on a prefilled 5 mL His60 Ni Superflow cartridge (Clontech Laboratories) at a flow rate of 5 mL min^−1^ in equilibration buffer (50 mm Tris‐HCl, 150 mm NaCl, pH 7.5).

Before elution of the hexahistidine tagged proteins by addition of 8 CV elution buffer (50 mm Tris‐HCl, 150 mm NaCl, 500 mm imidazole, pH 7.5), the column was washed with 8 CV equilibration buffer and 7 CV wash buffer (50 mm Tris‐HCl, 150 mm NaCl, 35 mm imidazole, pH 7.5).

Removal of imidazole and buffer exchange after IMAC was achieved by dialysis against sortase buffer (50 mm Tris‐HCl, 150 mm NaCl and 10 mm CaCl_2_, pH 7.5) using a cellulose ester membrane with a molecular weight cut‐off of 3.5–5 kDa (Spectrum Laboratories).

### Gel electrophoresis

Denaturing sodium dodecyl sulfate‐polyacrylamide gel electrophoresis (SDS‐PAGE) was carried out according to a standard protocol.^27^ For each gel, PageRuler Plus Prestained Protein Ladder (Thermo Fisher Scientific) was used as molecular weight ladder standard. After electrophoresis, gels were stained with PageBlue protein staining solution (Thermo Fisher Scientific) according to the manufacturer's instructions.

### Protein determination

Protein concentration was determined with the DC Protein Assay (Bio‐Rad Laboratories) according to the manufacture's microplate assay protocol using bovine serum albumin as protein standard.

### Preparation of cetuximab‐bispidine conjugate (5′)

Erbitux solution (4 mL) for infusion (5 mg mL^−1^, Merck KGaA) were purified using Zeba Spin Desalting Columns (7 K MWCO, 10 mL, Thermo Scientific), containing sodium bicarbonate saline buffer (50 mm NaHCO_3_, 150 mm NaCl, pH 8.5). For the conjugation reaction, 15 nmol of the purified antibody were incubated with 1.5 μmol of **5** for 4 h at 40 °C in sodium carbonate‐bicarbonate buffer (100 mm, pH 9.1)^28^ with gentle shaking. The reaction mixture was purified by size‐exclusion chromatography using Zeba Spin Desalting Columns (7 K MWCO, 5 mL, Thermo Scientific) with elution in 2‐(*N*‐morpholino)ethanesulfonic acid (MES)/NaOH buffer (100 mm, 150 mm NaCl, pH 6.5). The purified conjugate **5**′ was sterile filtered using Whatman Puradisc FP 30 cellulose acetate syringe filter units with a pore size of 0.2 μm (GE Healthcare Life Sciences) and stored at 4 °C.

### Preparation of sdAb‐bispidine conjugate (6′)

#### Sortase A‐mediated functionalisation

Small‐scale reactions were set up in 500 μL with variable molar rations of SrtA, sdAb and (Gly)_3_‐Lys‐N_3_ (Iris Biotech GmbH) and different incubation times. The optimal conditions and molar ratios (1:1:10) were upscaled and the reaction mixture was composed of 0.5 μmol SrtA, 0.5 μmol sdAb and 5 μmol (Gly)_3_‐Lys‐N_3_ in sortase buffer (50 mm Tris‐HCl, 150 mm NaCl and 10 mm CaCl_2_, pH 7.5). Bioconjugation reactions were incubated at 30 °C for up to 6 h with gentle shaking.

#### Purification of conjugation reactions

In the first purification step, all remaining hexahistidine tagged proteins were eliminated from the reaction mixture by IMAC using prepacked His60 Ni Gravity Columns (Clontech Laboratories). After collection of the flow‐through, the gravity‐flow column was washed twice with equilibration buffer (50 mm Tris‐HCl, 150 mm NaCl, pH 7.5). These wash fractions as well as the flow‐through were analysed for the presence of the azide‐functionalised sdAb conjugate by SDS‐PAGE.

The remaining unconjugated (Gly)_3_‐Lys‐N_3_ was removed in a second purification step by affinity chromatography using a Strep‐Tactin XT system (IBA Lifesciences) in combination with an ÄKTA pure chromatography system (GE Healthcare). The flow through as well as the wash fractions of the first purification step were loaded using an automated sample pump with a flow rate of 0.5 mL min^−1^. Affinity chromatography was performed on a prefilled 5 mL Strep‐Tactin XT Superflow cartridge (IBA lifesciences) at a flow rate of 5 mL min^−1^ in buffer W (100 mm Tris‐HCl, 150 mm NaCl, pH 8.0). Before elution of the Strep tagged proteins by addition of 8 CV elution buffer BXT (100 mm Tris‐HCl, 150 mm NaCl, 1 mm EDTA, 50 mm biotin, pH 8.0), the column was washed with 8 CV buffer W.

#### Strain‐promoted azide–alkyne cycloaddition

Small‐scale reactions were set up in 50 μL with variable molar ratios of azide‐functionalised sdAb and **6**. The optimal conditions and molar ratios (1:5) were upscaled and the reaction mixture was composed of 275 nmol azide‐functionalised sdAb and 1375 nmol of **6**. Reactions were incubated at 25 °C for up to 4 h with gentle shaking.

Non‐conjugated **6** was removed by size‐exclusion chromatography using Zeba Spin Desalting Columns (7 K MWCO, Thermo Scientific) with elution in MES/NaOH buffer (100 mm, 150 mm NaCl, pH 6.5). The purified conjugate **6**′ was sterile filtered using Whatman Puradisc FP 30 cellulose acetate syringe filter units with a pore size of 0.2 μm (GE Healthcare Life Sciences) and stored at 4 °C.

### Matrix‐assisted laser desorption ionisation time‐of‐flight (MALDI‐TOF) mass spectrometry

2,5‐Dihydroxyactetophenone (2,5‐DHAP, Bruker Daltonik) was used as matrix for MALDI‐TOF MS. For solubilisation of the matrix, 7.6 mg of 2,5‐DHAP were dissolved in 375 μL of absolute ethanol. After this, 125 μL of an 18 mg mL^−1^ aqueous solution of diammonium hydrogen citrate (Sigma–Aldrich) were added.

Protein samples were desalted by chloroform/methanol protein precipitation as described elsewhere.^29^ The dried protein pellets were dissolved in 50–100 μL 0.1 % TFA solution. A 2 μL aliquot of this desalted protein sample was mixed with 2 μL of 2 % TFA solution. After addition of 2 μL of matrix solution, the mixture was pipetted up and down until the crystallisation started and the solution became cloudy. Finally, 0.5 μL of the crystal suspension was spotted onto the ground steel target plate and the droplet was air‐dried completely at room temperature.

Spectra were acquired with an autoflex II TOF/TOF (Bruker Daltonik) in positive linear mode in combination with the flexControl software (Version 3.3, Bruker Daltonik) and analysed with the flexAnalysis software (Version 3.3, Bruker Daltonik). Theoretical molecular weights were calculated using the Compute pI/Mw tool on the ExPASy Server.

### ^64^Cu‐labelling experiments

The production of ^64^Cu was performed via proton irradiation of enriched ^64^Ni at a TR‐Flex cyclotron from Advanced Cyclotron Systems Inc (ACSI, Canada) and module‐assisted separation as described in detail recently.^30^ For ^64^Cu^II^ labelling, the pH of the [^64^Cu]CuCl_2_ solution was adjusted by adding one volume of 1 m MES/NaOH, pH 6.0. The radiochemical yield and purity were determined by radio‐HPLC and radio‐TLC. A solution of bispidine ligands **2**–**6** (40 nmol in 40 μL 100 mm MES/NaOH‐buffer of pH 6.5) spiked with 40 MBq [^64^Cu]CuCl_2_ was stirred for 30 min at room temperature. To determine the maximum molar activity, ^64^Cu‐labelling of bispidine ligands **2**–**6** was investigated in the concentration range of 0.1 to 10 nmol/200 μL MES‐NaOH buffer (pH 6.5), containing 100 MBq [^64^Cu]CuCl_2_ at different time points (5, 30, 60 min). The radiochemical yields were analysed using radio‐TLC. The results presented are the average of two independent experiments.

### Determination of distribution ratio log *D*_o/w_ at 25±1 °C

Log *D* ratios of ^64^Cu^II^‐labelled bispidine ligands **2**–**6** were determined using 1‐octanol/buffer mixtures. The experiments were performed with 100 μm solutions of bispidine ligands dissolved in aqueous buffer solutions. Aqueous phases consisted of 440 μL of 50 mm 4‐(2‐hydroxyethyl)‐1‐piperazine ethanesulfonic acid (HEPES)‐NaOH buffer (pH 7.2, 7.4, 7.6), 10 μL of [^64^Cu]CuCl_2_ solution (500 kBq) and 50 μL of a 100 μm Cu(NO_3_)_2_ solution. The distribution experiments were carried out at 25±1 °C in microcentrifuge tubes (2 cm^3^) with mechanical shaking for 30 min. The phase ratio *V*(_1‐octanol)_:*V*(_aq)_ was 1:1 (0.5 mL each). Full complexation was checked by radio‐TLC, which gave no evidence of free copper(II) in the aqueous phase. All samples were centrifuged and the phases then separated. The copper(II) complex concentration in both phases was determined radiometrically using γ‐radiation (^64^Cu, NaI(Tl) scintillation counter automatic gamma counter 1480, Wizard 3′′, Perkin–Elmer). The results are the average values of three independent experiments.

### Stability assessment by ligand challenge

Ligands **2**–**6** (40 nmol) were radiolabelled with 40 MBq [^64^Cu]CuCl_2_ in a total of 40 μL 100 mm MES‐NaOH, pH 6.5 at 25 °C for 30 min. The formation of the complexes were analysed by radio‐TLC using glass microfiber chromatography paper impregnated with silica gel (iTLC‐SG) as stationary phase and a 2 m NH_4_OAc/MeOH (1:2 *v*/*v*) mixture as mobile phase. The radiochemical yield was higher than 99.9 %, otherwise the labelling reaction was discarded. For ligand challenge, 10 nmol of the radiolabelled ligands (10 μL) were incubated with a 1000‐fold molar excess of the challenging ligands EDTA or DOTA, respectively, in 100 mm HEPES‐NaOH, pH 7.4 at 37 °C for up to 24 h. At each time point, the ratio between transchelated ^64^Cu as [^64^Cu]Cu‐EDTA or [^64^Cu]Cu‐DOTA (*R*_f_=1), respectively, and [^64^Cu]Cu‐bispidine (*R*_f_=≈0.7) was determined by radio‐TLC. The chromatography stripes were air‐dried and exposed to a high‐resolution phosphor‐imaging plate (GE Healthcare). The exposed plates were scanned with an Amersham Typhoon 5 Scanner (GE Healthcare), and the images were analysed using the ImageQuant TL software (Version 8.1, GE Healthcare).

### Radiolabelling of bispidine conjugates 5′ and 6′

For all radiolabelling procedures involving the bispidine‐functionalised proteins **5**′ and **6**′, a conjugate to [^64^Cu]CuCl_2_ ratio of 1:1 (nmol/MBq) was maintained. The reactions were set up in MES/NaOH buffer (100 mm, 150 mm NaCl, pH 6.5) and incubated at 37 °C for 30 min with gentle shaking.

For subsequent in vitro studies using [^64^Cu]Cu‐**6**′, this labelling reaction was upscaled. Accordingly, 100 nmol of **6**′ were radiolabelled with 100 MBq of [^64^Cu]CuCl_2_ in MES/NaOH buffer (100 mm, 150 mm NaCl, pH 6.5).

The extent of ^64^Cu^II^ complexation was assessed by radio‐TLC, using glass microfiber chromatography paper impregnated with silica gel (iTLC‐SG) as stationary phase and 2 m NH_4_OAc/MeOH (1:2 v/v) mixture as mobile phase. As a control, radio‐TLC analysis of [^64^Cu]Cu‐**6** was also performed under the same conditions. Individual solutions (5 μL) were first mixed with 5 μL of EDTA (500 mm, pH 7.4) and ≈1 μL was spotted at the origin. After TLC, the chromatography stripes were air‐dried and exposed to a high‐resolution phosphor‐imaging plate (GE Healthcare). The exposed plates were scanned with an Amersham Typhoon 5 Scanner (GE Healthcare). Free ^64^Cu^II^ (as EDTA complex) migrated to the solvent front (*R*_f_=1), whereas the radiolabelled proteins remained at the origin (*R*_f_=0). [^64^Cu]Cu^II^‐**6** has an *R*_f_ of ≈0.7. In addition to radio‐TLC, ^64^Cu^II^‐labelling of **6**′ was confirmed by SDS‐PAGE and subsequent radioluminography.

If necessary, the radiolabelled proteins were purified prior to further use via spin filtration with Amicon Ultra‐0.5 centrifugal filter devices with a molecular weight cutoff of 3 K (Amicon Ultra 3 K device, Merck‐Millipore).

### Cell culture

Cell culture flasks, dishes and plates (CELLSTARS) were supplied by Greiner Bio‐One GmbH. The adherent human tumour cell lines A431 (ATCC number: CRL‐1555 was maintained as previously reported.^22^, ^31^ The cell line was confirmed to be mycoplasma‐negative using the Venor GeM Advance Mycoplasma Detection Kit (Minerva Biolabs) and was tested monthly.

### Confocal microscopy

A total of 100 000 A431 cells were seeded in 35 mm imaging dishes (IBIDI) in 2 mL DMEM supplemented with 10 % foetal calf serum (FCS), and incubated in a humidified atmosphere of 95 % air/5 % CO_2_ at 37 °C. After 24 h of incubation, the medium was refreshed and cells were incubated with 100 nm of **6**′ at 37 °C for up to 24 h. Afterwards, cells were washed thrice with ice‐cold PBS, fixed with 4 % paraformaldehyde and 2.5 % sucrose in PBS, and permeabilised with 0.25 % TritonX‐100 in PBS for 10 min. To prevent unspecific antibody binding, cells were incubated with 10 % FCS in PBS overnight at 4 °C. Cells were then incubated with rabbit anti‐EGFR (D38B1) Alexa Fluor 647 monoclonal antibody (Cell Signaling Technology) and with StrepMAB‐Classic Chromeo 488 conjugate (IBA Lifesciences) for 2 h at room temperature in the dark. Cells were again washed three times with PBS, and the nuclei were stained using Hoechst 33258. Fluorescence microscopy was performed with a Fluoview 1000 confocal laser scanning microscope (Olympus) using a 60× (NA 1.35) oil objective.

### Cell binding studies

A total of 40 000 A431 cells were seeded in 48‐well plates in 250 μL DMEM supplemented with 10 % foetal calf serum (FCS) and incubated in a humidified atmosphere of 95 % air/5 % CO_2_ at 37 °C. After 48 h of incubation, the medium was replaced by serum‐free DMEM and cultivation was continued for ≈4 h. The plates were pre‐incubated for 30 min at 4 °C before the addition of different concentrations of [^64^Cu]Cu^II^‐6′ ranging from 12 pm to 400 nm. To control wells only, an excess of unlabelled sdAb (4 μm final concentration) was added to determine the non‐specific binding. The cell culture microplates were further incubated for 1 h at 4 °C. Following treatment with [^64^Cu]Cu^II^‐6′, cells were washed three times with ice‐cold PBS to ensure removal of loosely attached proteins from the cell membrane. Finally, cell lysis was achieved by the addition of 1 % SDS/0.1 m NaOH and incubation for 30 min at room temperature with vigorous shaking. The cell‐associated radioactivity was quantified using an automated gamma counter (Perkin–Elmer Life and Analytical Sciences). Total protein concentration in cell extracts was determined as described above. The equilibrium dissociation constant *K*_d_ as well as the maximum specific binding *B*_max_ were determined from the measured data using the Prism software (Version 7, GraphPad).

### PET studies

In vivo experiments were performed at the Semmelweis University Department of Biophysics and Radiation Biology according to the National Institute of Food Chain Safety (Licence No.: XIV‐I‐001/29‐7/2012 and PE/EA/50‐2/2019). Naval Medical Research Institute (NMRI)‐Foxn1^nu/nu^ mice were used for the in vivo experiments and were purchased from JANVIER LABS (JANVIER LABS, Saint‐Berthevin Cedex, France). A431 cells were grown in culture as described earlier. For the pre‐clinical experiments of tumour binding in PET diagnostics, one million A431 cells in 0.2 mL of medium were injected to the right flank of the mice. Four weeks after tumour cell inoculation, tumours of circa 3 mm in diameter were formed. Mice were then used for the PET imaging dynamic study to establish the tumour binding kinetics of [^64^Cu]Cu^II^‐**6**′. Therefore, approximately 2 nmol with 39 MBq of the respective protein were injected into a lateral tail vein of NMRI‐Foxn1^nu/nu^ mice. This was followed by dynamic PET scans for 2 h and repetitive scans after 1.7, 16.7, 41.7 and 63.3 h using a microPET P4 scanner (Siemens). Data evaluation was carried out using the ROVER software (ABX GmbH). Quantitative time activity curves were expressed as mean of the standardised uptake value (SUV) ± SEM. SUV was estimated by the following equation: [tissue concentration (MBq mL^−1^)×the body weight (g)]/injected dose (MBq).

## Conflict of interest

The authors declare no conflict of interest.

## Supporting information

As a service to our authors and readers, this journal provides supporting information supplied by the authors. Such materials are peer reviewed and may be re‐organized for online delivery, but are not copy‐edited or typeset. Technical support issues arising from supporting information (other than missing files) should be addressed to the authors.

Supplementary
