# Supplementary material for: Versatile Bispidine‐Based Bifunctional Chelators for 64CuII‐Labelling of Biomolecules
Source: Chemistry. 2020 Jan 9;26(9):1989–2001. doi: 10.1002/chem.201904654 (PMC7028042; doi:10.1002/chem.201904654)
Supplement: Supplementary file 1 — Supplementary [file CHEM-26-1989-s001.pdf]

CHEMISTRY
A European Journal

Supporting Information

Versatile Bispidine-Based Bifunctional Chelators for $^{64}Cu^{II}$-Labelling of Biomolecules

Garima Singh,[a] Kristof Zarschler,*[a] Sebastian Hunoldt,[a] Irma Ivette Santana Martínez,[a] Carmen L. Ruehl,[b] Madlen Matterna,[a] Ralf Bergmann,[a] Domokos Máthé,[c, d] Nikolett Hegedüs,[c] Michael Bachmann,[a] Peter Comba,*[b] and Holger Stephan*[a]

chem_201904654_sm_miscellaneous_information.pdf

Table of contents:

Figure S1. ^{1}H NMR (A), ^{13}C NMR (B) and characteristic chemical shifts of protons of *anti*- and *syn*-product **of 1**

Figure S2. ^{1}H NMR (A), ESI-MS (B) and FT-IR (C) of **2**

Figure S3. ^{1}H NMR (A), ^{13}C NMR (B), ESI-MS (C) and FT-IR (D) of **3**.

Figure S4. ^{1}H NMR (A), ESI-MS (B) and FT-IR (C) of **4**.

Figure S5. ^{1}H NMR (A), ^{13}C NMR (B), ESI-MS (C) and FT-IR (D) of **5**.

Figure S6. ^{1}H NMR (A), ESI-MS (B) and FT-IR (C) of **6**.

Figure S7. Radio-TLC analysis of ligand exchange reactions in presence of EDTA (A) and DOTA (B) as competing ligand (1000-fold excess) after 24 h at 37°C.

Figure S8. MALDI-TOF mass spectra of cetuximab before (A) and after (B) conjugation with **5.**

Figure S9. Thin-layer chromatography of ^{64}Cu-labelled bispidine-conjugated cetuximab. Radiolabelled **5'** remains at the origin (R_f = 0), whereas ([^{64}Cu]Cu-EDTA)$^{2-}$ migrates with the solvent front (R_f = 1). Noteworthy, any excess of [^{64}Cu]$CuCl_2$ is complexed by the addition of EDTA.

Figure S10. SDS-PAGE analysis of sortase-mediated azide-functionalisation of the single-domain antibody (sdAb) derivative 7C12-Strep-sortag-6HIS. The molar amount of $(Gly)_3$-Lys-N_3 was varied (50-500 nmol) while the amount of the sdAb as well as the enzyme was kept constant (50 nmol).

Figure S11. Radio-TLC analysis of ^{64}Cu-labelled bispidine-conjugated sdAb. After conjugation of the chelating agent, the conjugate **6'** was labelled with [^{64}Cu]$CuCl_2$ and analysed by radio-TLC. Radiolabelled **6'** remains at the origin (R_f = 0), whereas [^{64}Cu]Cu-EDTA migrates with the solvent front (R_f = 1). The reference [^{64}Cu]Cu-**6** has an R_f of ~0.7. Noteworthy, any excess of [^{64}Cu]$CuCl_2$ was complexed prior to TLC by the addition of EDTA.

Figure S12. Confocal immunofluorescence microscopy images of A431 cells exposed to **6'** for up to 24 h showing specific binding and co-localisation of the immunoconjugate with EGFR. A431 cells were incubated with 100 nM **6'** for the indicated periods at 37°C. Binding of the immunoconjugates to plasma membrane localised EGFR was analysed by indirect immunofluorescence. To visualise the expression of EGFR, an anti-EGFR Alexa Fluor® 647 antibody conjugate was used (A; red fluorescence). An anti-Strep-tag Chromeo™488 conjugate was used to detect the Strep-tagged **6'** (B; green fluorescence). The nuclei were visualised by the DNA binding stain Hoechst 33258 (C; blue fluorescence). The overlay of (A), (B) and (C) is shown in (D). Scale bars = 20 µm.

Table S1. Stability assessment of [^{64}Cu]Cu-labelled bispidine derivatives by radio-challenge experiments. Radiolabelled substances were incubated for the indicated period in the presence of 1000-fold molar excess of the challenging ligands EDTA or DOTA, respectively. Following radio-TLC, the amount (%) of [^{64}Cu]Cu-EDTA or [^{64}Cu]Cu-DOTA formed was determined**.**

Table S2. List of the expected average molecular masses as well as observed m/z values of the singly-charged and doubly-charged molecular ions for each protein derivative.

A)

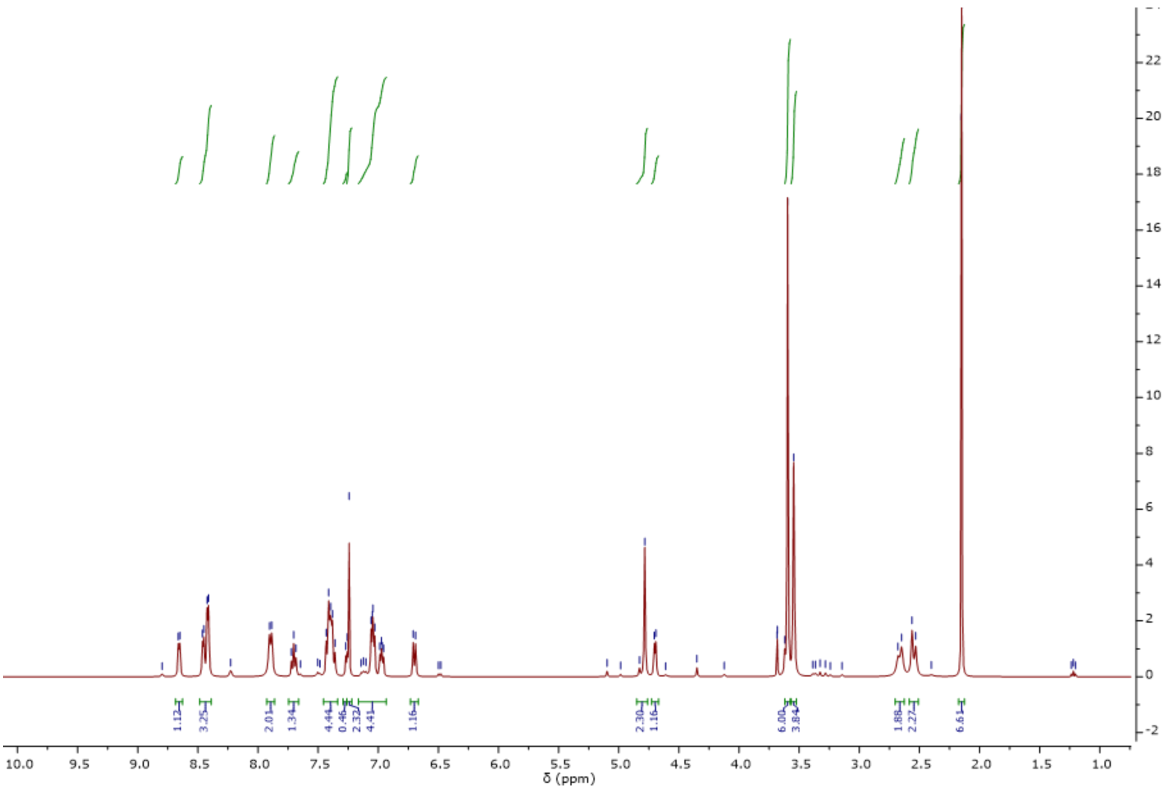

B)

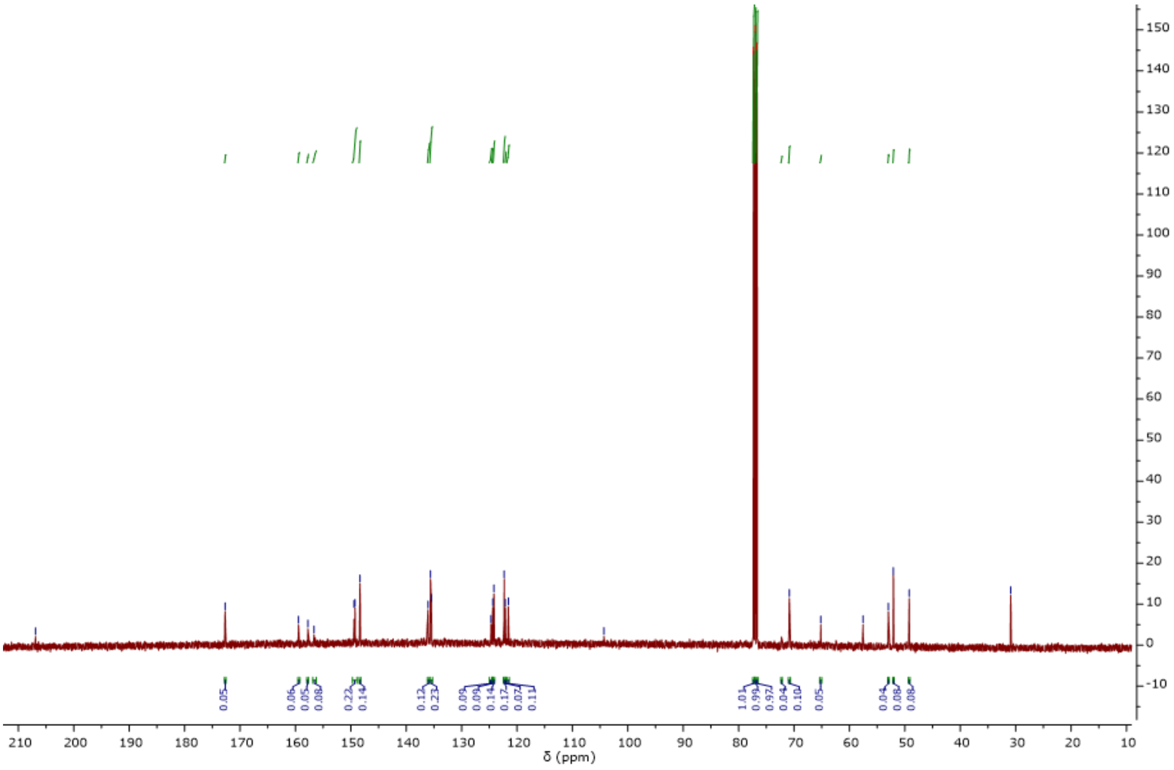

C)

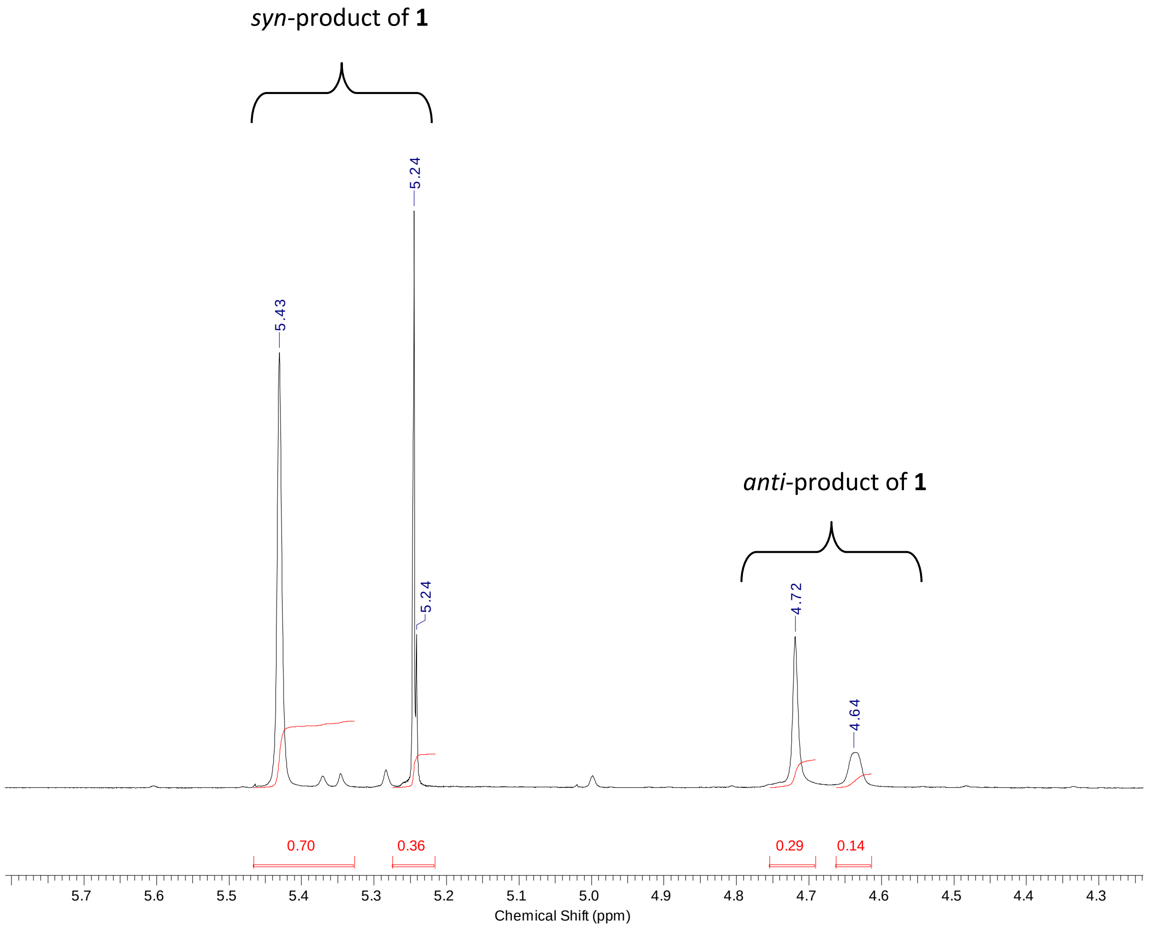

Figure S1. ^{1}H NMR (A), ^{13}C NMR (B) and characteristic chemical shifts of protons of *anti*- and *syn*-product* of **1**.

* reduction of bispidine with $NaBH_4$ in dry methanol yields 70 % *syn*- and 30 % *anti*-product; reduction in dioxane/water (3/2 = v/v) exclusively yields the *anti*-product.

A)

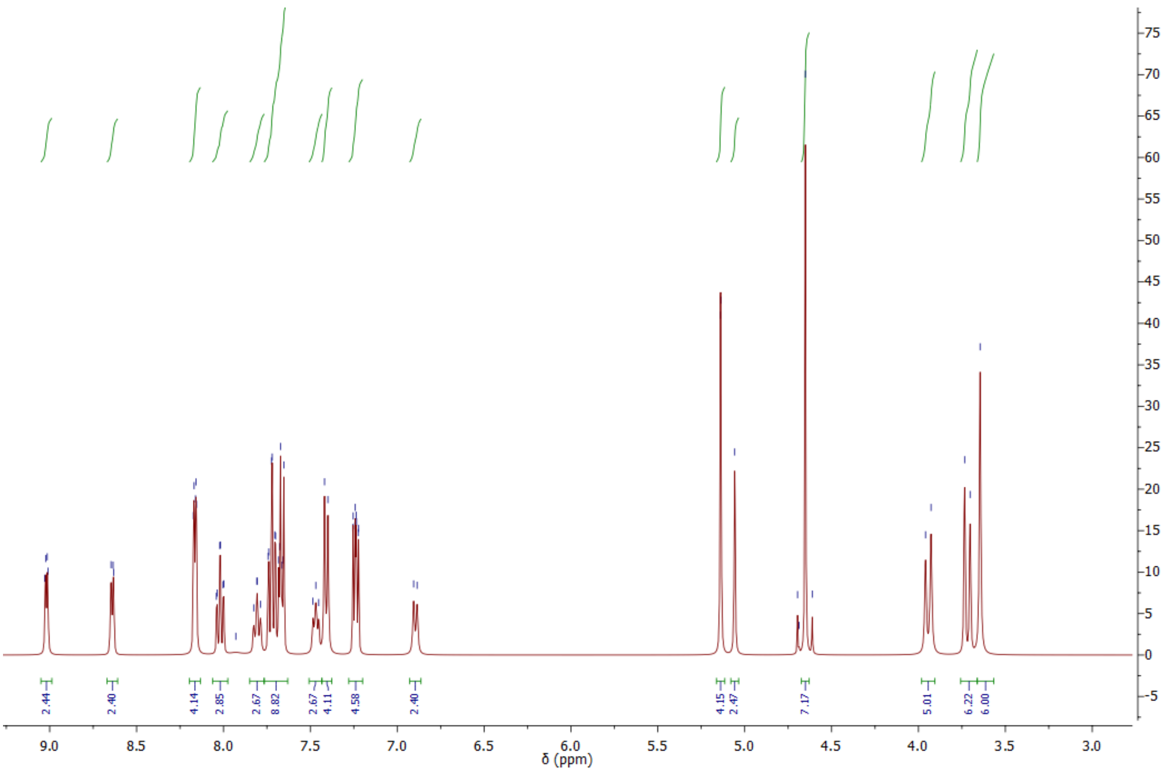

B)

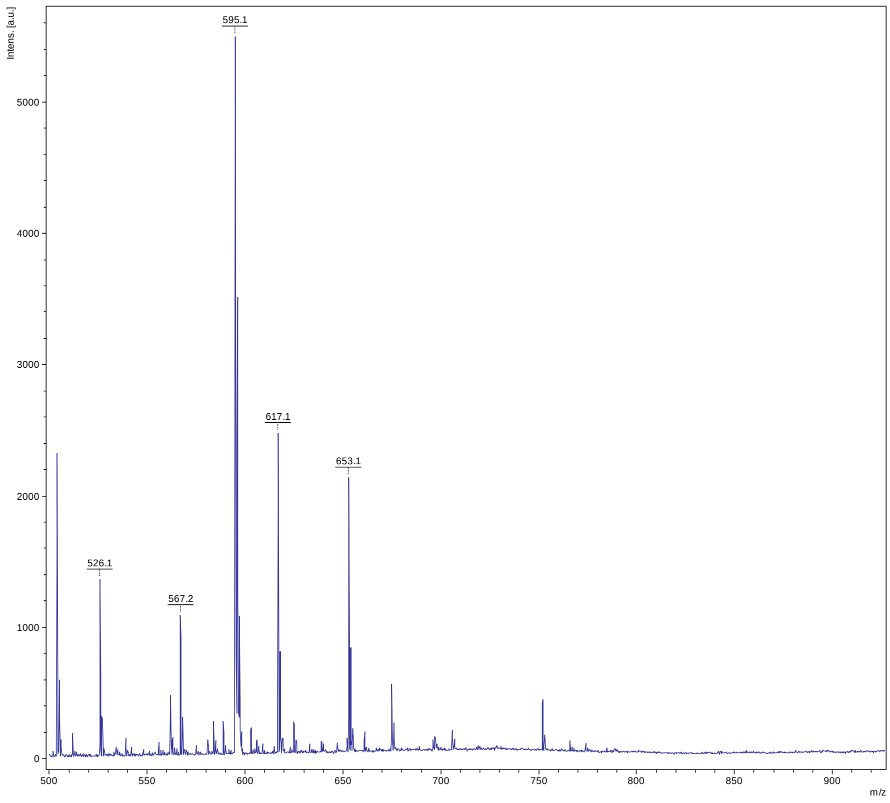

C)

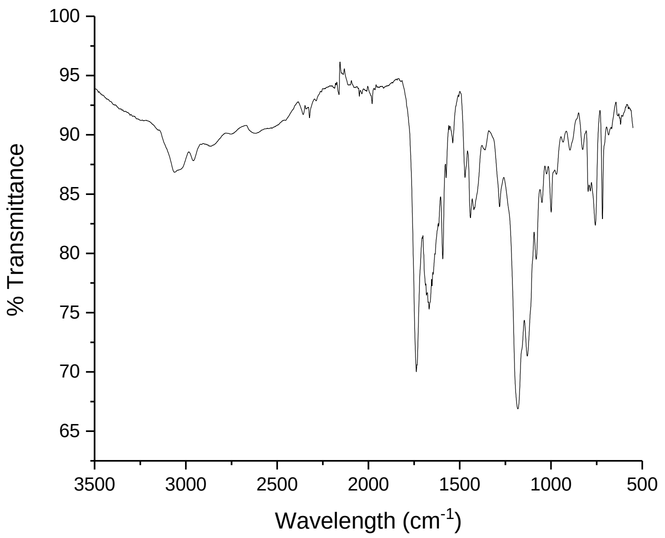

Figure S1. ^{1}H NMR (A), ESI-MS (B) and FT-IR (C) of **2**.

A)

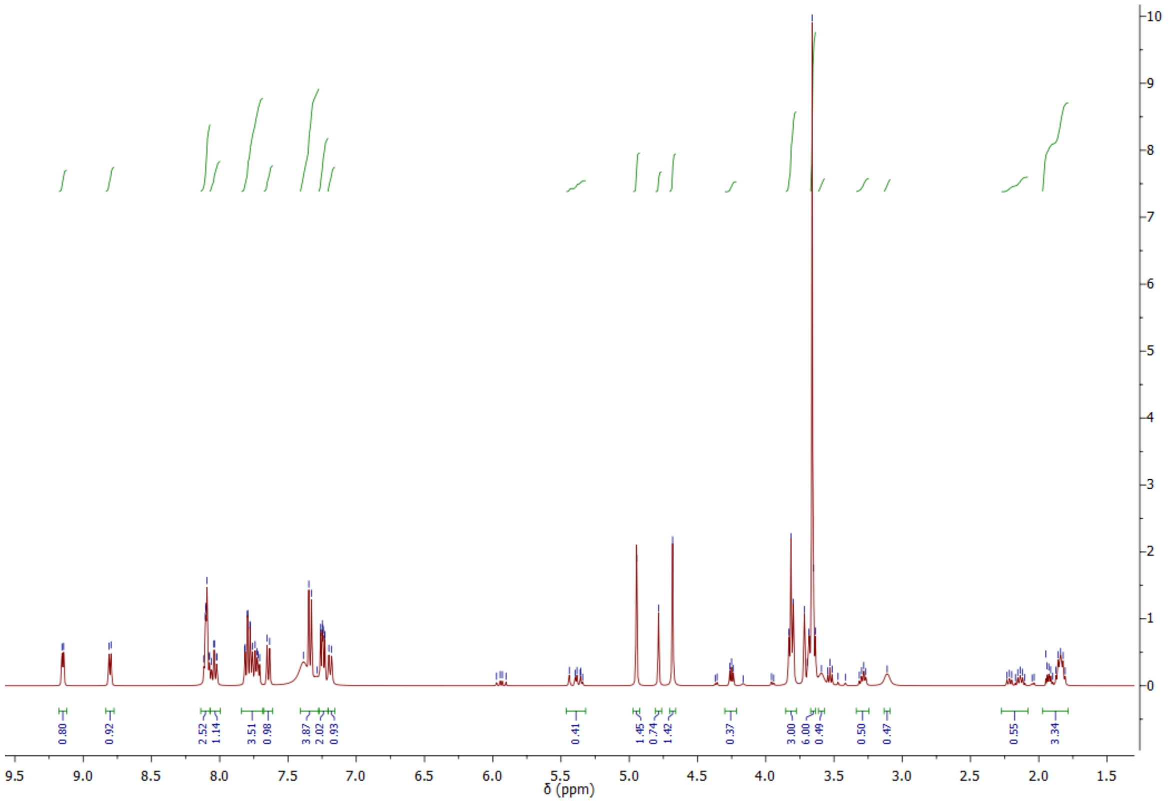

B)

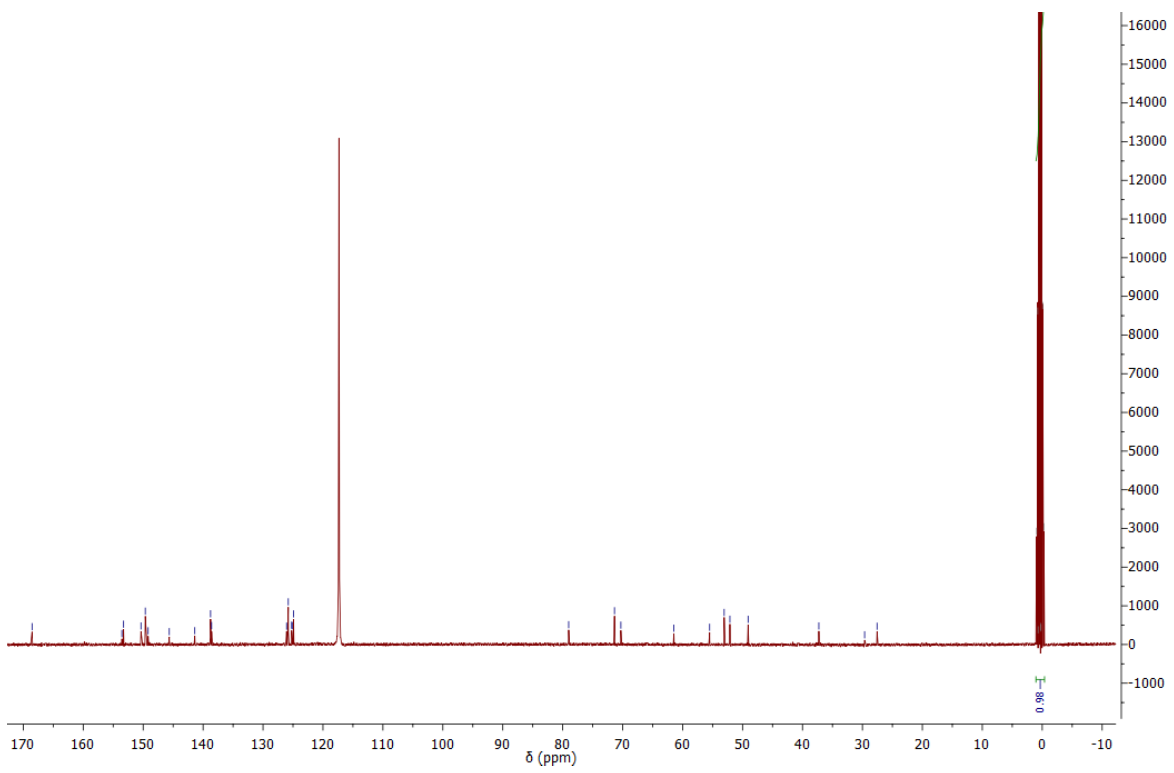

16000
15000
14000
13000
12000
11000
10000
9000
8000
7000
6000
5000
4000
3000
2000
1000
0
-1000
0.98
170 160 150 140 130 120 110 100 90 80 70 60 50 40 30 20 10 0 -10
δ (ppm)

C)

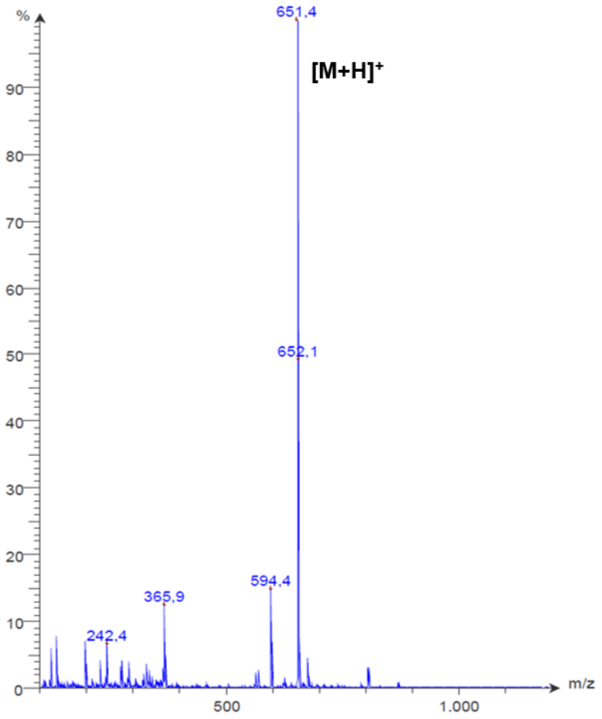

%
651,4
[M+H]+
652,1
594,4
365,9
242,4
90
80
70
60
50
40
30
20
10
0
500
1.000
m/z

D)

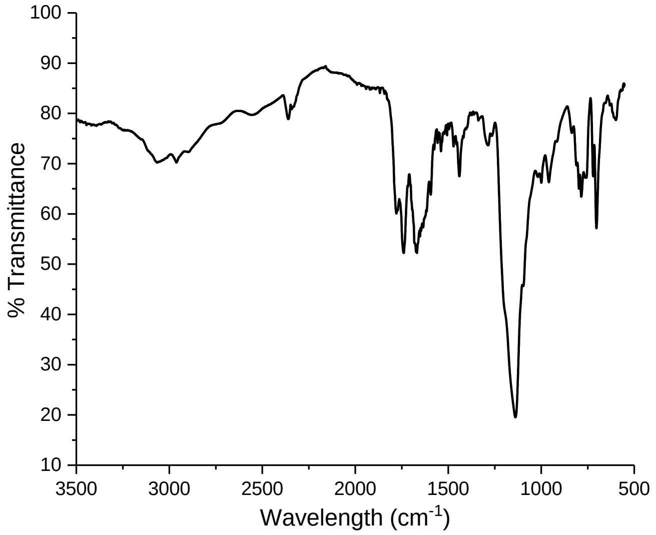

Figure S2. ^{1}H NMR (A), ^{13}C NMR (B), ESI-MS (C) and FT-IR (D) of **3**.

A)

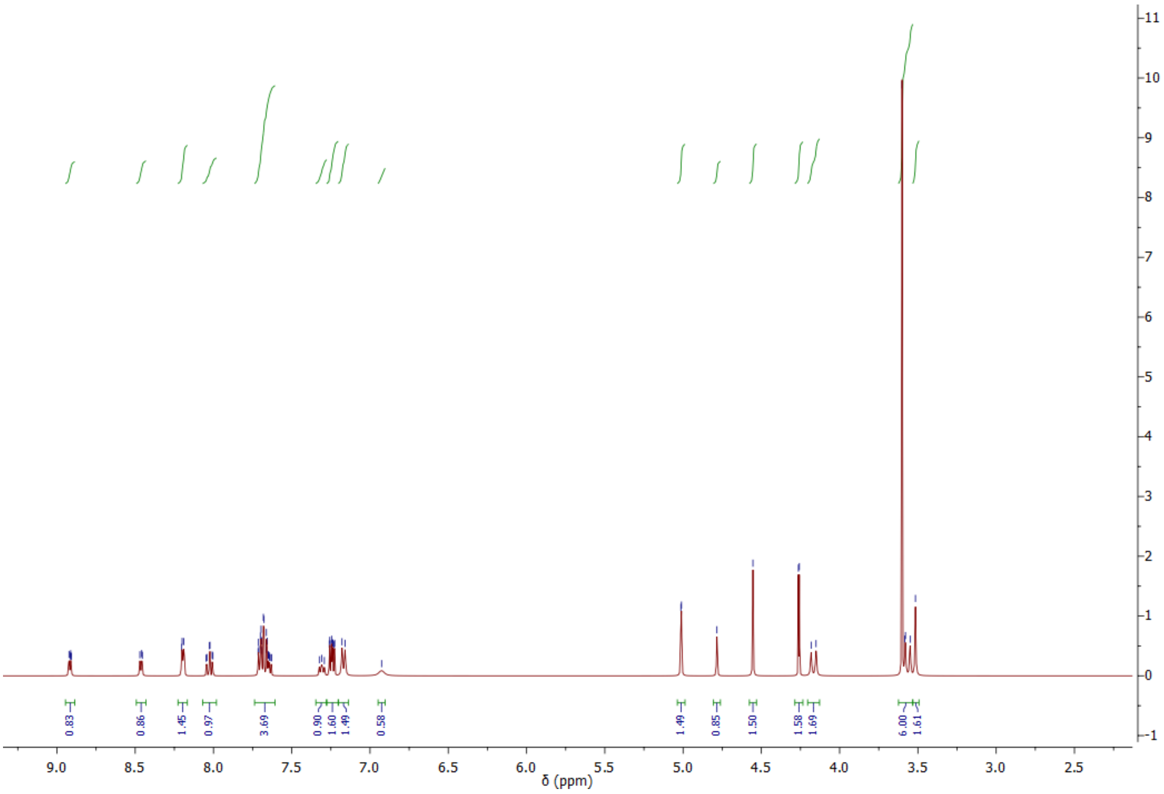

B)

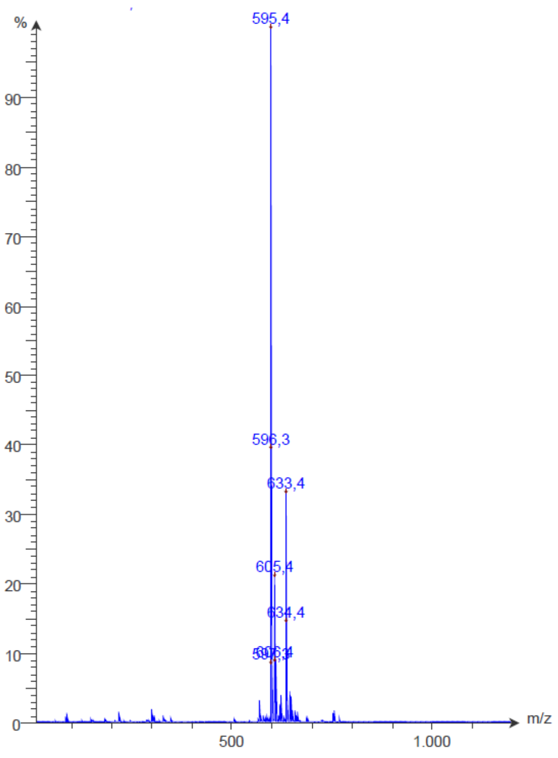

C)

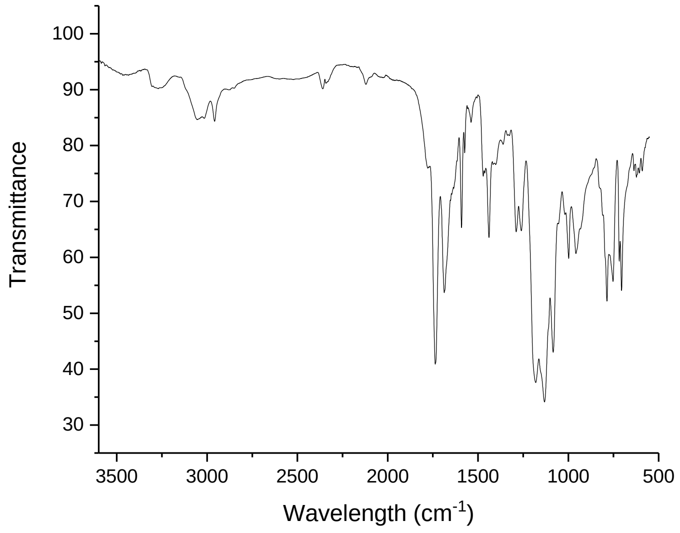

Figure S3. ^{1}H NMR (A), ESI-MS (B) and FT-IR (C) of **4**.

A)

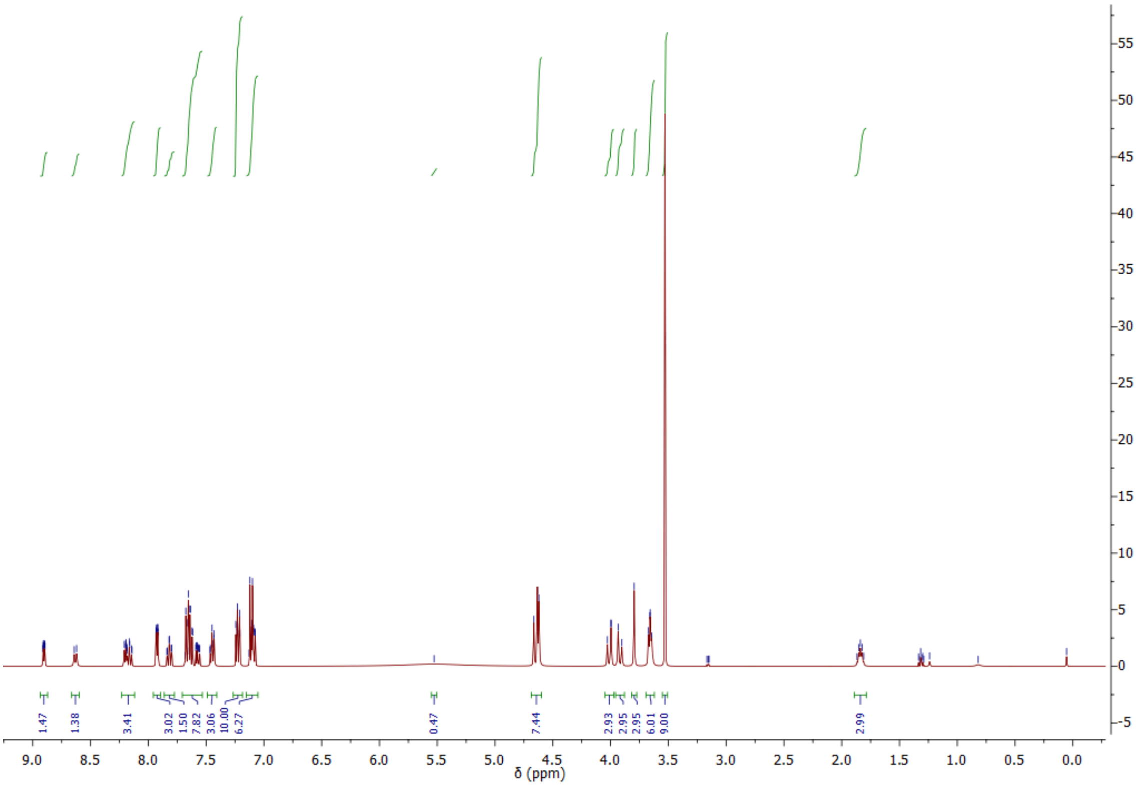
1.47
1.38
3.41
3.02
1.50
7.82
3.06
10.00
6.27
0.47
7.44
2.93
2.95
2.95
6.01
9.00
2.99
δ (ppm)

B)

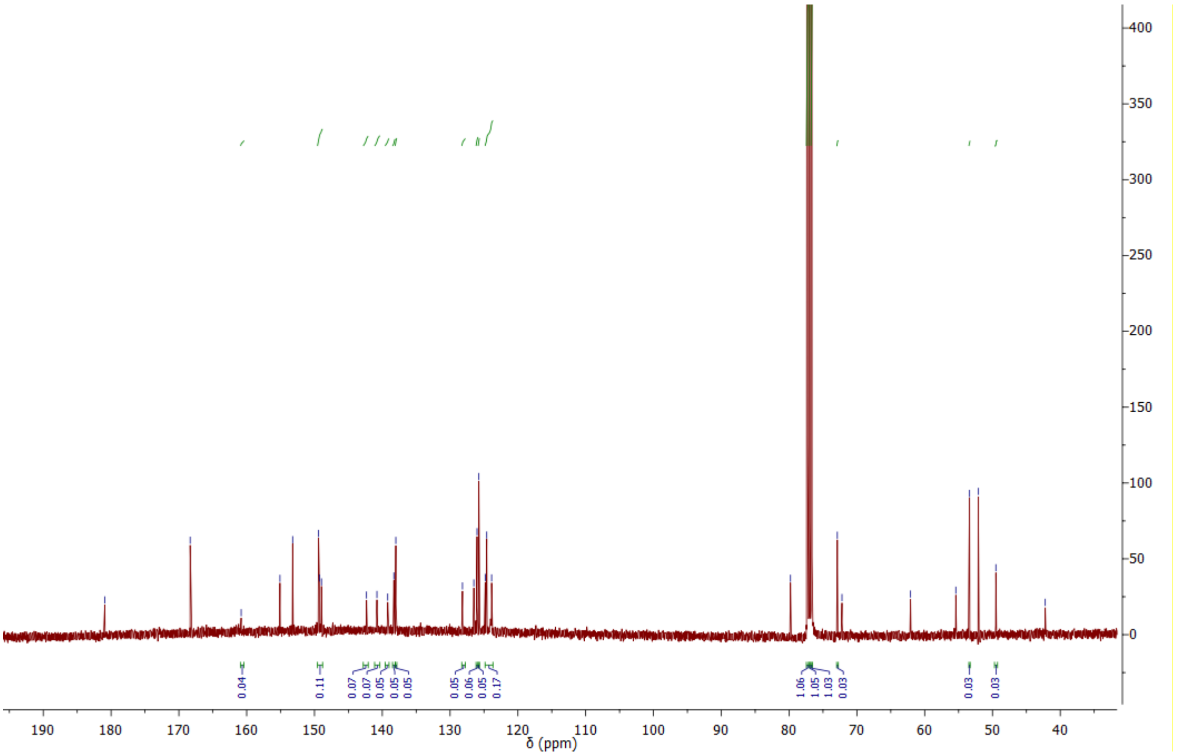
0.04
0.11
0.07
0.07
0.05
0.05
0.05
0.05
0.06
0.05
0.17
1.06
1.05
1.03
0.03
0.03
0.03
δ (ppm)

C)

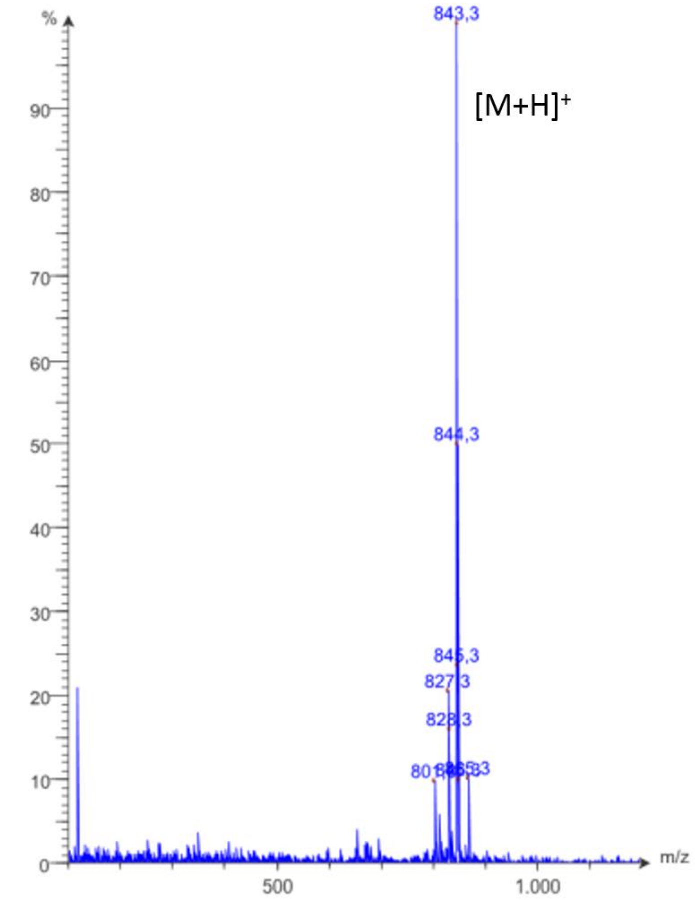

D)

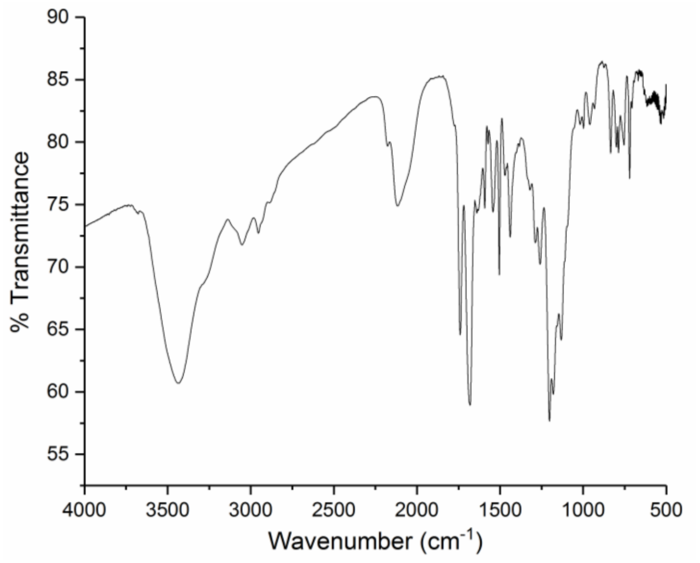

Figure S4. ^{1}H NMR (A), ^{13}C NMR (B), ESI-MS (C) and FT-IR (D) of **5**.

A)

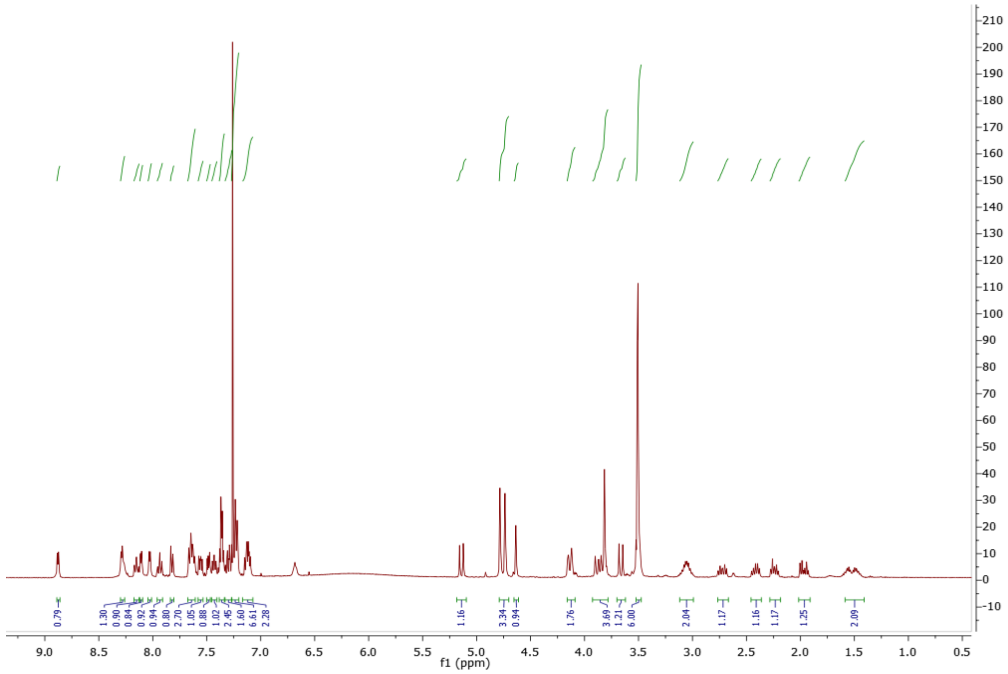

B)

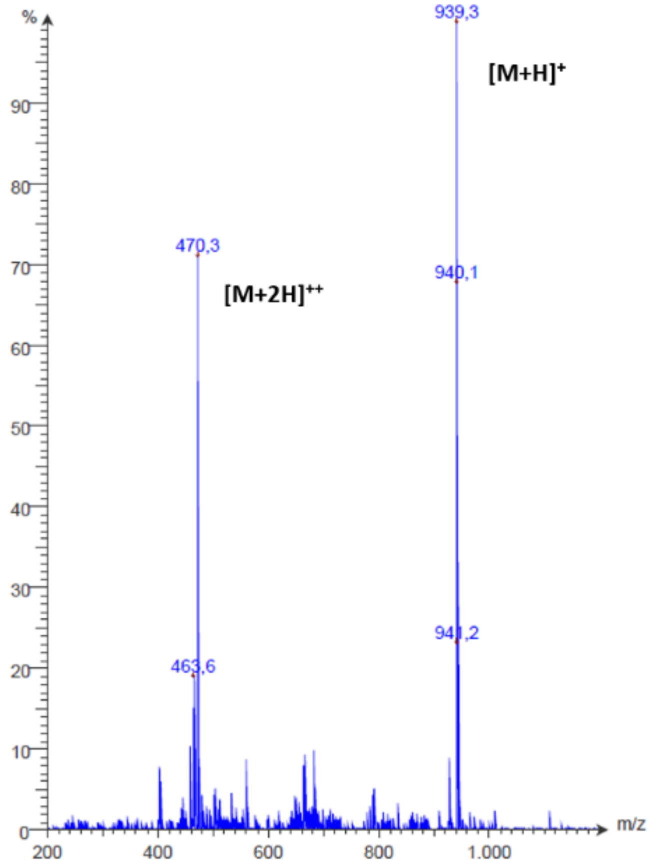

C)

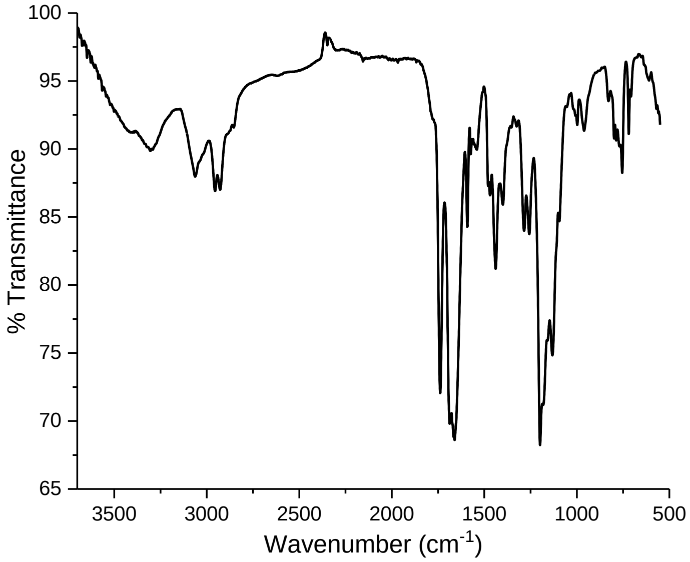

Figure S5. ^{1}H NMR (A), ESI-MS (B) and FT-IR (C) of **6**.

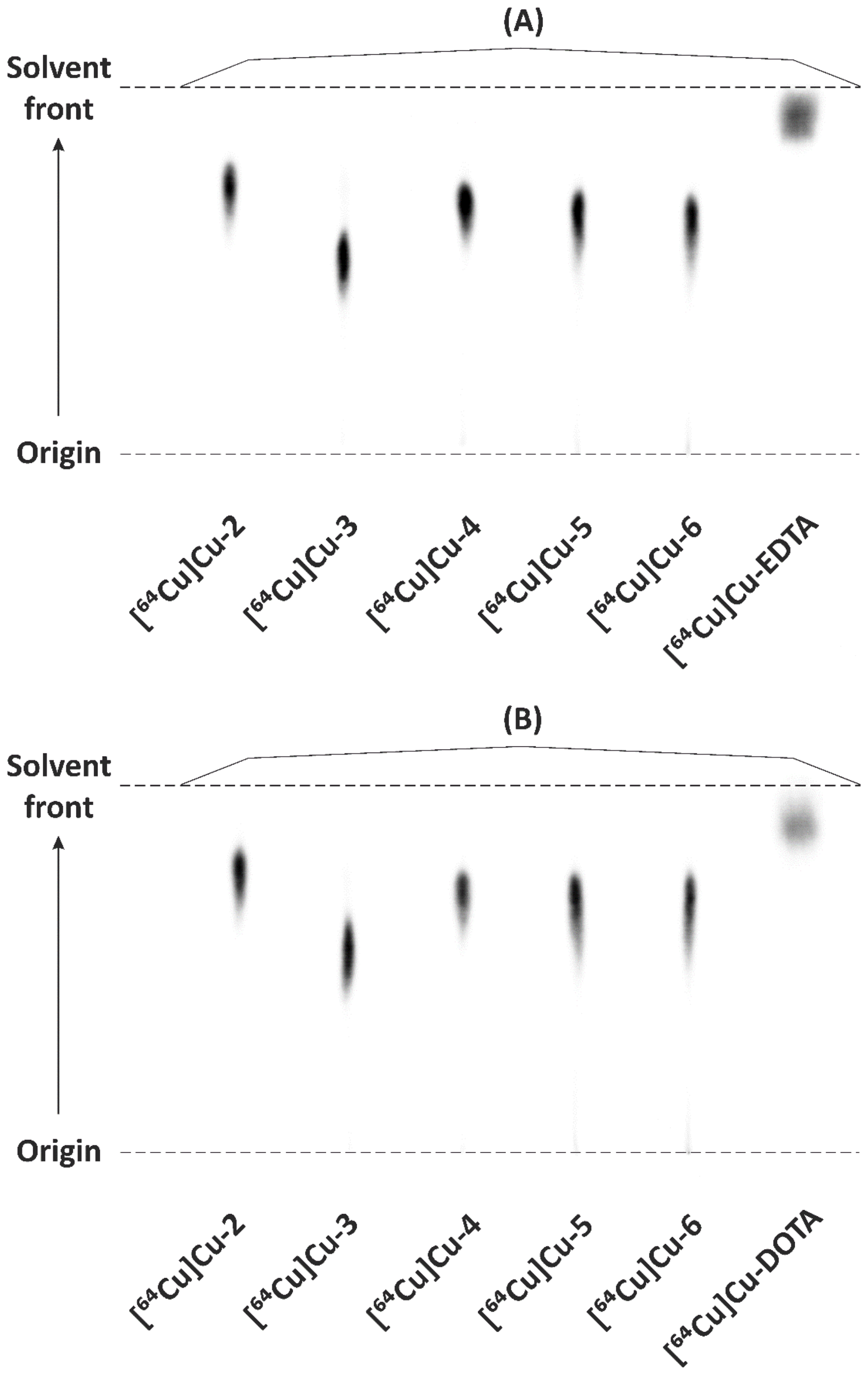

Figure S6. Radio-TLC analysis of ligand exchange reactions in presence of EDTA (A) and DOTA (B) as competing ligand (1000-fold excess) after 24 h at 37°C.

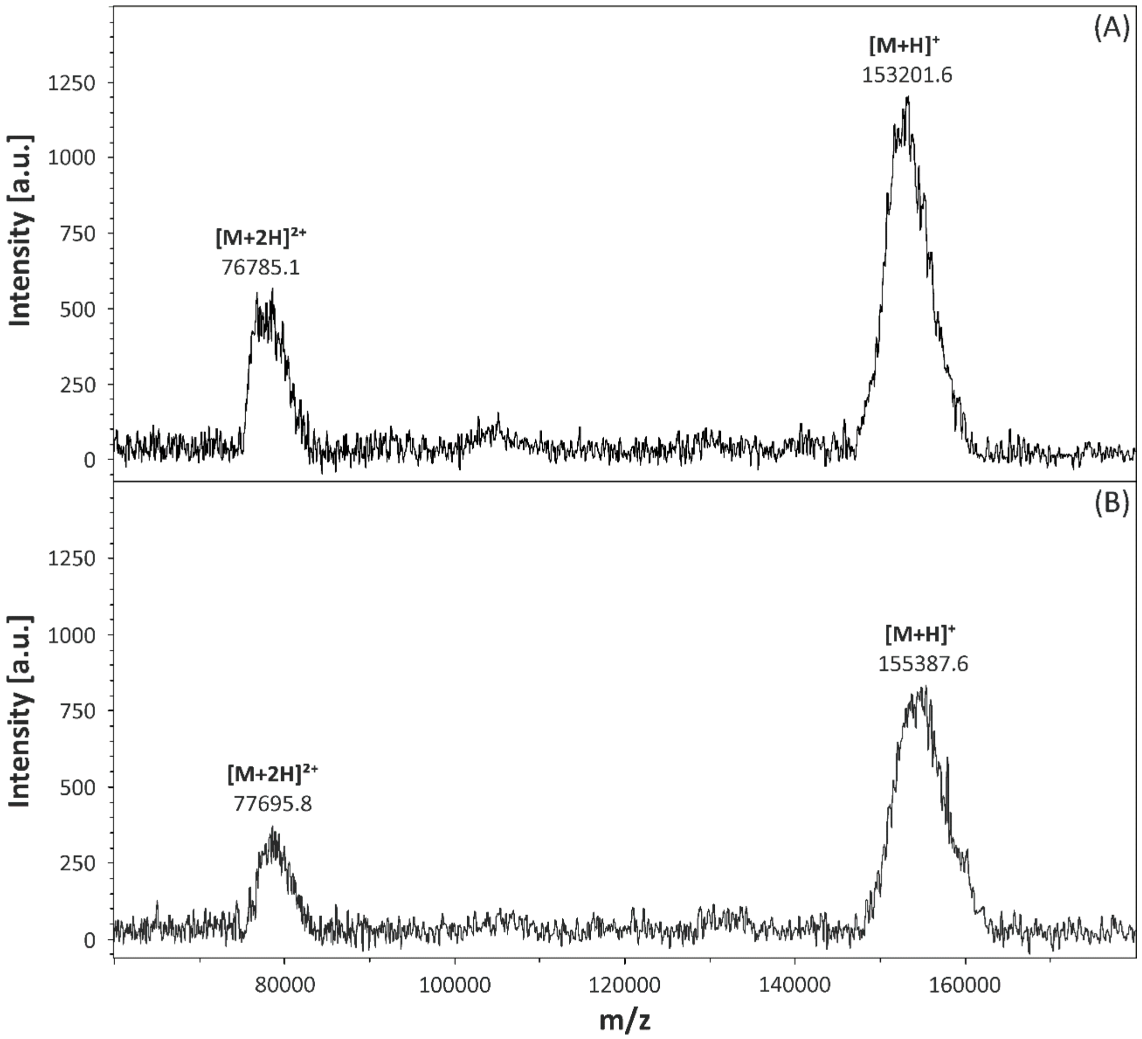

Figure S7. MALDI-TOF mass spectra of cetuximab before (A) and after (B) conjugation with **5.**

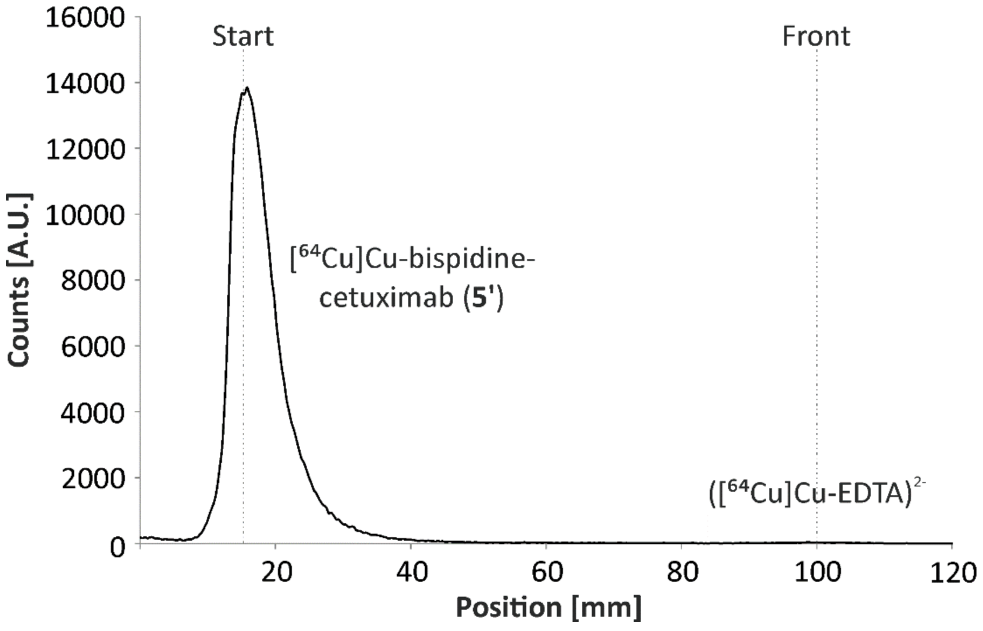

Figure S8. Thin-layer chromatography of ^{64}Cu-labelled bispidine-conjugated cetuximab. Radiolabelled **5'** remains at the origin (R_f = 0), whereas ([^{64}Cu]Cu-EDTA)$^{2-}$ migrates with the solvent front (R_f = 1). Noteworthy, any excess of [^{64}Cu]CuCl$_2$ is complexed by the addition of EDTA.

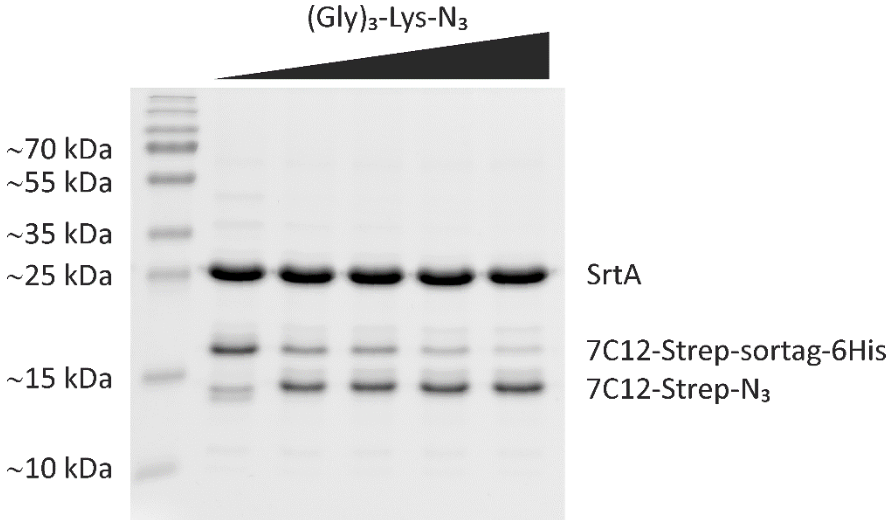

Figure S9. SDS-PAGE analysis of sortase-mediated azide-functionalisation of the single-domain antibody (sdAb) derivative 7C12-Strep-sortag-6HIS. The molar amount of $(Gly)_3$-Lys-N_3 was varied (50-500 nmol) while the amount of the sdAb as well as the enzyme was kept constant (50 nmol).

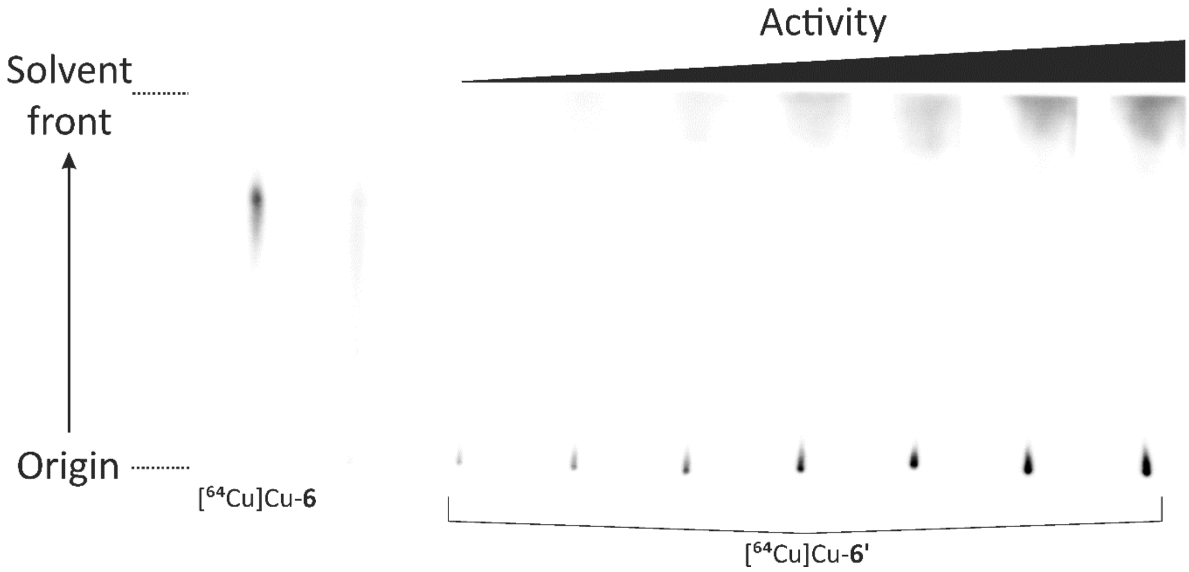

Figure S10. Radio-TLC analysis of ^{64}Cu-labelled bispidine-conjugated sdAb. After conjugation of the chelating agent, the conjugate **6'** was labelled with [^{64}Cu]$CuCl_2$ and analysed by radio-TLC. Radiolabelled **6'** remains at the origin (R_f = 0), whereas [^{64}Cu]Cu-EDTA migrates with the solvent front (R_f = 1). The reference [^{64}Cu]Cu-**6** has an R_f of ~0.7. Noteworthy, any excess of [^{64}Cu]$CuCl_2$ was complexed prior to TLC by the addition of EDTA.

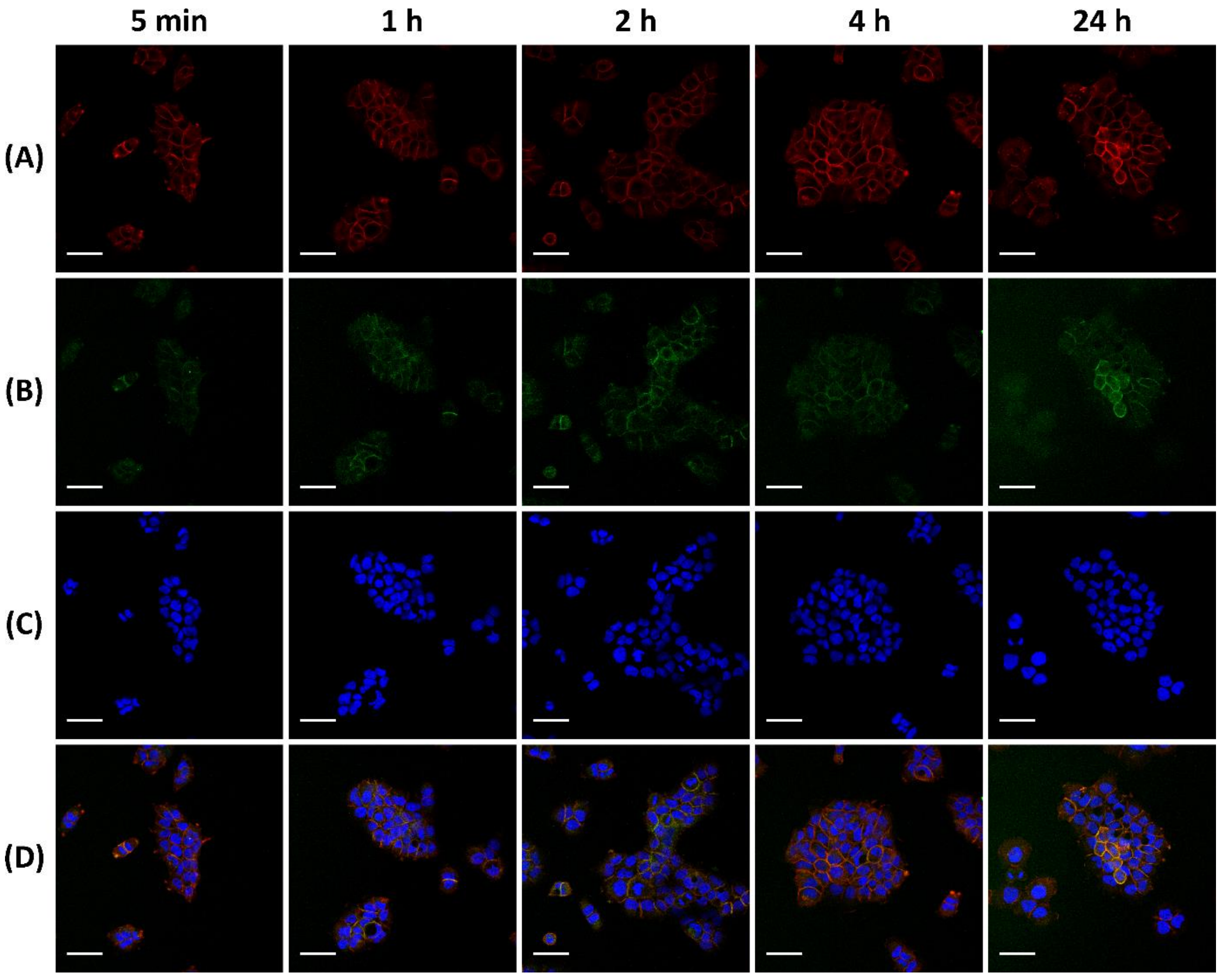

Figure S11. Confocal immunofluorescence microscopy images of A431 cells exposed to **6’** for up to 24 h showing specific binding and co-localisation of the immunoconjugate with EGFR. A431 cells were incubated with 100 nM **6’** for the indicated periods at 37°C. Binding of the immunoconjugates to plasma membrane localised EGFR was analysed by indirect immunofluorescence. To visualise the expression of EGFR, an anti-EGFR Alexa Fluor® 647 antibody conjugate was used (A; red fluorescence). An anti-Strep-tag Chromeo™488 conjugate was used to detect the Strep-tagged **6’** (B; green fluorescence). The nuclei were visualised by the DNA binding stain Hoechst 33258 (C; blue fluorescence). The overlay of (A), (B) and (C) is shown in (D). Scale bars = 20 µm.

Table S1. Stability assessment of [^{64}Cu]Cu-labelled bispidine derivatives by radiochallenge experiments. Radiolabelled substances were incubated for the indicated period in the presence of 1000-fold molar excess of the challenging ligands EDTA or DOTA, respectively. Following radio-TLC, the amount (%) of [^{64}Cu]Cu-EDTA or [^{64}Cu]Cu-DOTA formed was determined.

	**Incubation time**			
**EDTA challenge**	**1 h**	**2 h**	**4 h**	**24 h**
Bispidine-acetic acid **2**	0.030	0.028	0.018	0.034
Bispidine-amine **3**	0.048	0.023	0.029	0.054
Bispidine-alkyne **4**	0.038	0.031	0.024	0.044
Bispidine-isothiocyanate **5**	0.034	0.006	0.010	0.073
Bispidine-DBCO **6**	0.044	0.025	0.032	0.055

	**Incubation time**			
**DOTA challenge**	**1 h**	**2 h**	**4 h**	**24 h**
Bispidine-acetic acid **2**	0.019	0.010	0.025	0.012
Bispidine-amine **3**	0.018	0.029	0.024	0.015
Bispidine-alkyne **4**	0.025	0.018	0.018	0.016
Bispidine-isothiocyanate **5**	0.003	0.011	0.009	0.010
Bispidine-DBCO **6**	0.026	0.025	0.022	0.013

Table S2. List of the expected average molecular masses as well as observed m/z values of the singly-charged and doubly-charged molecular ions for each protein derivative.

**Protein / Conjugate**	**Calculated molecular mass**	**Observed m/z**	
		$[M+H]^+$	$[M+2H]^{2+}$
7C12-Strep-sortag-6HIS	18428.9	18322.4	9153.3
7C12-Strep-N_3	16646.2	16551.9	8236.3
**6’**	17584.4	17444.9	8721.7
